# Variations in salivary microbiome and metabolites are associated with immunotherapy efficacy in patients with advanced NSCLC

**DOI:** 10.1128/msystems.01115-24

**Published:** 2025-02-10

**Authors:** DanHui Huang, YueHua Chen, Cui Li, Shuang Yang, LiShan Lin, XiaoNan Zhang, XiaoFang Su, LaiYu Liu, Haijin Zhao, Tingyue Luo, Shaoxi Cai, QianNan Ren, Hangming Dong

**Affiliations:** 1 Chronic Airways Diseases Laboratory, Department of Respiratory and Critical Care Medicine, Nanfang Hospital, Southern Medical University 662307 , Guangzhou, Guangdong, China; 2 Department of Radiation Oncology, Nanfang Hospital, Southern Medical University 70570 , Guangzhou, Guangdong Province, China; Kobenhavns Universitet, Frederiksberg, Denmark

**Keywords:** saliva microbiome, fat metabolism, immunotherapy response, non-small cell lung cancer

## Abstract

Lung cancer is a leading cause of cancer mortality, with non-small cell lung cancer (NSCLC) comprising the majority of cases. Despite the advent of immune checkpoint inhibitors (ICIs), a significant number of patients fail to achieve a durable response, highlighting the need to understand the factors influencing treatment efficacy. Saliva samples and tumor samples were collected from 20 NSCLC patients. The salivary microbiota was profiled using metagenomic next-generation sequencing, and metabolites were analyzed via liquid chromatography-mass spectrometry to identify correlations among bacteria, metabolites, and immunotherapy responses. Immunohistochemistry (IHC) analysis of tissue samples verified the result. Besides, *in vitro* experiments and tumor tissue microarray, including 70 NSCLC patients, were utilized to further explore the potential mechanism linking the oral microbiome and immunotherapy efficacy. The study revealed several differential species and distinct metabolite compositions between responders and non-responders to ICI therapy in NSCLC and explored correlations and mechanisms between microbiota metabolites and immunotherapy resistance. Notably, it was found that several *Neisseria* and *Actinomyces* species were significantly enriched in responders and identified lipids and lipid-like molecules associated with PD-L1 expression levels and treatment outcomes. Importantly, several differential lipid molecules were associated with differential species. Further, *in vitro* experiments and IHC experiments indicated that abnormal fat metabolism linked to dysbiosis is correlated with immunotherapy resistance through regulation of CD8^+^ T cell activity/infiltration and PD-L1 expression. Specific saliva microbiome and its associated lipids metabolites are significantly associated with the efficacy of ICI-based therapy in lung cancer. Our findings suggest that oral microbiome modulation and targeting lipid metabolism could improve immunotherapy responses, offering new avenues for personalized treatment strategies.

**IMPORTANCE:**

In non-small cell lung cancer, our study links specific salivary microbiome profiles and related lipid metabolites to the efficacy of immune checkpoint inhibitor (ICI) therapies. Responders showed enrichment of certain *Neisseria* and *Actinomyces* species and distinct lipid compositions. These lipids correlate with PD-L1 expression and CD8^+^ T cell activity, affecting treatment outcomes. Our results imply that modulating the oral microbiome and targeting lipid metabolism may enhance ICI effectiveness, suggesting novel personalized therapeutic approaches.

## INTRODUCTION

Lung cancer remains a significant public health concern, being the primary cause of global cancer mortality with an estimated 125,070 deaths in the United States in 2024 (1). Over the past decade, there have been substantial advancements in the treatment of lung cancer. Enhanced understanding of lung cancer biology has led to the development of targeted therapies and immunotherapy. Immune checkpoint inhibitors (ICIs) are now first-line therapies for advanced non-small cell lung cancer (NSCLC), yet nearly 70% of patients do not experience lasting benefits, highlighting the need to understand the factors influencing treatment efficacy (2). Therefore, it is crucial to investigate the mechanism of immunotherapy resistance and search for potential predictive markers.

The human body’s surface barriers are home to complex communities of bacteria. The functions encoded within the human microbiome play a vital role in various aspects of human physiology. The oral microbiome stands as the second most diverse habitat within the human body, following only behind the gut microbiome (3). The oral microbiota interacts with the immune system of its host, which plays an important role not only in oral cavity health but also in systemic health, including lung diseases (3). For instance, periodontal disease linked to microbes including *Porphyromonas gingivalis* has been reported to be associated with lung cancer (4). Recent prospective studies have found that lower α diversity in *salivary microbiome* was to higher risk of developing lung cancer (5, 6).

Furthermore, mounting evidence suggests that oral microbiota can influence and modulate host immune responses. For example, oral bacteria, particularly those associated with periodontitis, have been linked to systemic diseases such as cardiovascular disease, diabetes, and respiratory infections. These bacteria and their by-products can modulate immune responses beyond the oral cavity, potentially promoting systemic conditions (7, 8). Given the close connection between the oral cavity and the lungs, it is unsurprising that the oral microbiome can influence lung immune responses. This idea is supported by multiple animals and clinical studies. For example, primo-colonizing oral bacteria can influence lung epithelial cells by inducing cytokine production and gene expression changes, thereby impacting lung immunity and morphology (9). Episodic aspiration with oral commensals induces a prolonged Th17 response that decreases susceptibility to respiratory pathogens (10). Therefore, it is plausible that the oral microbiome were associated with the immunotherapy efficacy of lung cancer patients. However, specific distribution characteristics and mechanisms of oral microbiota in immunotherapy-sensitive and immunotherapy-resistant lung cancer patients remain unclear.

To address these assumptions, a case-control study was conducted to link information on salivary microbiota and metabolite data using metagenomic next-generation sequencing (mNGS) and non-targeted metabolomics-based liquid chromatography-mass spectrometry (LC/MS). We report the ICIs-based therapy efficacy in 20 advanced lung cancer patients and correlate clinical responses with salivary microbiota taxonomic profiles and salivary metabolite levels. Through multi-omics analysis, we found the important regulatory role of specific oral species distribution in immunotherapy resistance and proved its related fat metabolism is highly enriched in immunotherapy resistance lung cancer patients. Additionally, through expanding lung cancer patient sample size and conducting *in vitro* experiments, we confirmed that lung cancer immunotherapy resistance is closely associated with abnormal fat metabolism and verified its regulation on the infiltration of CD8^+^ T cell and the expression of checkpoint PD-L1 in lung cancer patients received immunotherapy. Thus, this study certifies and emphasizes the value of the oral microbiome as an immunoregulatory factor in lung cancer and identifies its associated fat metabolites involved in immune signaling pathways, which not only provides further understanding of oral microbiome in the occurrence of lung cancer but also holds promising potential as a guide for clinical treatment and prognosis analysis targeting lung cancer patients.

## MATERIALS AND METHODS

### Patient recruitment

Twenty newly diagnosed NSCLC patients with stage IIIB–IV at the Department of Respiratory and Critical Care Medicine, Nanfang Hospital, Southern Medical University (Guangzhou, China), from July 2021 to September 2022, were enrolled in this study. The inclusion criteria were as follows: (i) diagnosis of advanced non-small cell lung cancer patients was confirmed through tissue pathological biopsy and imaging; (ii) no anti-tumor treatment was performed before sampling, including surgical treatment, chemotherapy, radiotherapy, targeted therapy, immunotherapy, traditional Chinese medicine treatment, and so on; (iii) aged 18–79 years old; (iv) patients with PS score = 0–1; and (v) did not receive antibiotics within 1 month. The exclusion criteria were as follows: (i) patients with combined primary malignant tumor history of other systems; (ii) patients with acute oral infection, acute lung infection, chronic obstructive pulmonary disease with acute exacerbation, bronchial asthma, bronchiectasis with infection, active tuberculosis, and other acute infectious diseases within 4 weeks; or with serious heart, respiratory, kidney, and liver dysfunction; (iii) patients who used oral hormone, immunosuppressive agent or microbial preparation in the past 4 weeks; (iv) patients with irregular cycles of anti-tumor therapy; (v) lung adenocarcinoma patients with EGFR, ALK, ROS1, BRAF V600, NTRK, and MET exon 14 mutation. All enrolled participants received immunotherapy combined with chemotherapy as first-line therapy. All participants had measurable disease severity by computed tomography or magnetic resonance imaging using immune-modified Response Evaluation Criteria In Solid Tumors criteria (ImRECIST) (11). Responses to the treatment were evaluated every two treatment cycles and iUPDs were confirmed by a subsequent assessment no less than 4 weeks from the date first documented. Patients in this study were defined as responder (R) (with sustained complete remission, partial remission or disease stability at the time point of first cycle therapy for more than 9 months) or non-responder (NR) (with progression disease at the time point of first cycle therapy within 9 months).

### Sample collection and storage

All subjects were instructed to thoroughly brush their teeth and were subsequently prohibited from consuming any food or undertaking any oral hygiene measures until the collection of saliva samples the following morning. Saliva samples were collected from each participant in the morning before brushing (12) and placed into a 20 mL disposable sterile sample cup before therapy. Approximately 5 mL of saliva was obtained from each individual. Saliva did not contain impurities such as blood, food residue, phlegm, and so on. Otherwise, resampling was necessary. Saliva samples were collected before the first cycle of anti-tumor therapy. For patients in the NR group, saliva samples were collected again when the evaluation of tumor treatment efficacy indicated progressive disease (PD). The samples were stored in a refrigerator at −80°C until further processing.

### Nucleic acid extraction and sequencing

Saliva samples were further transferred to Dian Diagnostics Group Co., Ltd., Guangzhou, China for further analysis. DNA was extracted from all samples using a Pathogen DNA Kit (TIANamp Bacteria DNA Kit [DP302-02, TIANGEN, Beijing, China]) following the manufacturer’s instructions. DNA concentration was calibrated using the Qubit reagent (Q33230, ThermoFisher Scientific, Invitrogen, Carlsbad, CA, USA). Fragmentation Mix (FRM) reaction solution (SQK-RPB004 Rapid PCR Barcoding Kit [Nanopore], Oxford Nanopore Technologies, Oxford, UK) was prepared in a 0.2 mL PCR tube, stirred mildly, and mixed evenly, at 30°C for 5 min, 80°C for 1 min, and cooled quickly on an ice box. After the PCR product was transferred to a 1.5 mL centrifuge tube, an equal amount of 50 µL purified magnetic beads were supplied, mixed well, let stand for 5 min at room temperature, and placed on a magnetic stand after instantaneous centrifugation. When the solution turned clear, the supernatant was discarded. Following two cycles of washing with 180 µL of 80% ethanol, the sample was instantaneously centrifuged at 3,000 rpm for 1 min, placed on a magnetic stand to absorb the residual ethanol, dried at room temperature for 30 s with the lid uncovered, supplied with 10 µL of 10 mM Tris-HCl (pH = 8.0, PHG0002, SAFC Biosciences, Lenexa, KS, USA)and 50 mM NaCl (S8210, Solarbio Life sciences, Beijing, China) mixed solution, and gently rotated the suspended magnetic beads. Following incubation for 2 min at room temperature, the sample was placed on the magnetic stand. When the solution turned clear, the eluate was aspirated for later use. Qubit reagent was used to calibrate the concentration of each sample eluate. As per relevant concentrations, the same absolute quantity template was employed and mixed into 10 µL with a total concentration ranging from 100 to 200 ng/µL, supplemented with 1 µL Rapid Adapter (RAP) solution (SQK-RPB004 Rapid PCR Barcoding Kit [Nanopore], Oxford Nanopore Technologies, Oxford, UK), mixed gently, and incubated at room temperature for 15 min. Sequencing was performed when the loading mixture was ready within 15 min. The sequencing data volume was 500 Mb.

### Nanopore sequencing and microbiota data analysis

Raw data files were generated by the MinION sequencer (Oxford Nanopore Technologies, Oxford, UK) in fast5 format, and the real-time identification and the fastq files were completed using MinKnow software (version 1.11.5, Oxford Nanopore Technologies, Oxford, UK). Low-quality sequences were filtered using MinKnow software. Filtered data were removed from host DNA by Minimap2 software (version 2.17.r941, Broad Institute, Cambridge, MA, USA) using the human genome reference sequence Hg38). Sequencing data multi-sequence alignment and identification of pathogenic microorganisms sequencing data filtered by data and stripped of host DNA were performed with Centrifuge v1.0.3 (http://www.ccb.jhu.edu/software/centrifuge/, Center for Computational Biology, Johns Hopkins University, Baltimore, MD, USA) and nonredundant nucleic acid database at the National Center for Biotechnology Information. The species counts and relative abundance tables for the site were input into R-base V.4.1.0 for statistical analysis. For α diversity, we chose ACE index, Chao index, and Simpson and Shannon indexes for evaluation. For β diversity analysis, we estimated using Bray-Curtis distance and Jaccard distance and visualized by principal coordinate analysis (PCoA). Differential taxonomy was identified by linear discriminant analysis effect size (LEfSe) analysis at https://huttenhower.sph.harvard.edu/lefse/ (13).

### LC/MS analysis

UHPLC-MS/MS analyses were performed using a Vanquish UHPLC system (Thermo Fisher, Germany) coupled with an Orbitrap Q Exactive TMHF mass spectrometer (Thermo Fisher, Germany) in Novogene Co., Ltd. (Beijing, China). Samples were injected onto a HypesilGoldcolumn (100 × 2.1 mm^2^, 1.9 µm) using a 17 min linear gradient at a flow rate of 0.2 mL/min. The eluents for the positive polarity mode were eluent A (0.1% FA in water) and eluent B (methanol). The eluents for the negative polarity mode were eluent A (5 mM ammonium acetate, pH 9.0) and eluent B (methanol). The solvent gradient was set as follows: 2% B, 1.5 min; 2–85% B, 3 min; 85–100% B, 10 min; 100–2% B, 10.1 min; and 2% B, 12 min. Q Exactive HF mass spectrometer was operated in positive/negative polarity mode with spray voltage of 3.5 kV, capillary temperature of 320°C, sheath gas flow rate of 35 psi, aux gas flow rate of 10 L/min, S-lens RF level of 60, and Aux gas heater temperature of 350°C.

### Data processing and metabolite identification

The raw data files generated by UHPLC-MS/MS were processed using the Compound Discoverer 3.1 (CD3.1; ThermoFisher) to perform peak alignment, peak picking, and quantitation for each metabolite. The main parameters were set as follows: retention time tolerance, 0.2 min; actual mass tolerance, 5 ppm; signal intensity tolerance, 30%; signal/noise ratio, 3; and minimum intensity, and so on. After that, peak intensities were normalized to the total spectral intensity. The normalized data were used to predict the molecular formula based on additive ions, molecular ion peaks, and fragment ions. And then peaks were matched with the mzCloud at https://www.mzcloud.org, mzVault and MassList database to obtain the accurate qualitative and relative quantitative results. Statistical analyses were performed using the statistical software R (R version R-3.4.3), Python (Python 2.7.6version), and CentOS (CentOS release 6.6).

### Metabolite data analysis

These metabolites were annotated using HMDB database and LIPIDMaps database. Orthogonal partial least squares discriminant analysis (OPLS-DA) was performed using the OmicStudio tools at https://www.omicstudio.cn/tool (14). We applied Mann-Whitney *U* test to calculate the statistical significance (*P* value). PLS-DA was conducted to calculate the VIP score. Metabolites with VIP of >1, *P* value of <0.05, and fold change of ≥1.5 or ≤0.67 were considered to be differential metabolites. For clustering heat maps, the data were normalized using *z*-scores of the intensity areas of differential metabolites and were performed at https://www.omicstudio.cn/tool (14).

For additional materials and methods, see the supplemental material.

## RESULTS

### Patient characteristics

A total of 20 newly diagnosed NSCLC patients with stage IIIB–IV were enrolled in the study. The demographics and clinical characteristics of these 20 patients are listed in Table S1. All patients were followed up until disease progression or death, with the last follow-up conducted on 3 January 2023. Patients were grouped according to their clinical assessment, with 10 patients being evaluated as R (immunotherapy responder), with the others (*n* = 10) experiencing disease progression within 9 months after the first cycle of cancer treatment being classified as NR (immunotherapy non-responder). The 20 NSCLC patients were predominantly male with an average age of approximately 60 years. No significant difference was observed in age, body mass index, disease history, disease stage, smoking history, antibiotics usage, pathological type, and clinical stages among the patient cohort.

### Bacterial composition and diversity between NR and R groups

**Fig 1:**
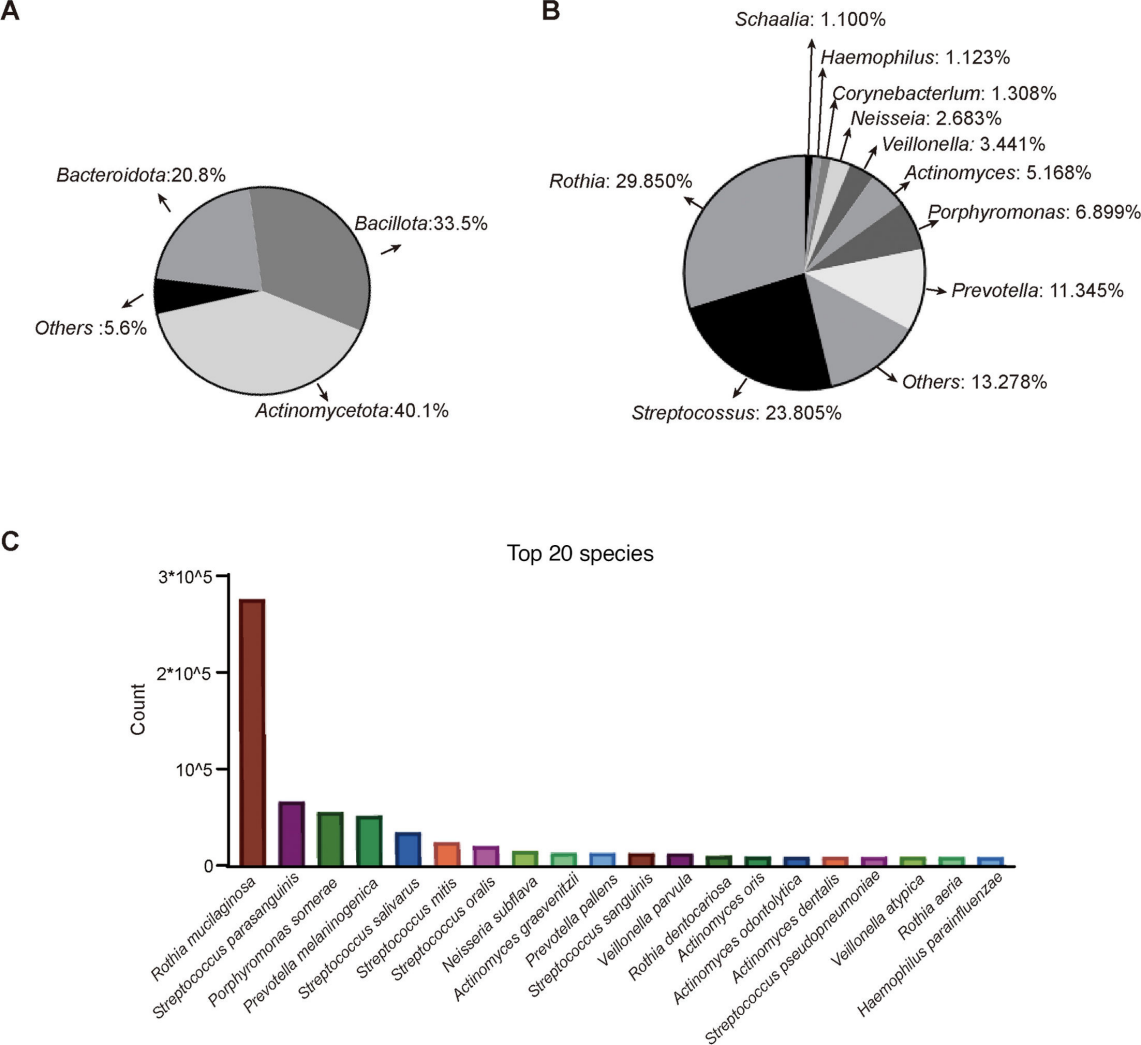
Salivary microbiome composition in lung cancer patients. (**A**) Main phylum among lung cancer patients was shown. (**B**) Main genera distribution among lung cancer patients were shown. (**C**) Top 20 oral bacterial species distribution among lung cancer patients were shown.

In general, through deep metagenome sequencing, saliva samples of 20 patients identified 3433 pathogenic bacteria at the species level. The sequence reads of each saliva sample were normalized to 1 million. The compositions of the top taxonomies in all participants are shown (Fig. 1A). At the phylum level, the most abundant phyla were *Actinomycetota*, *Bacillota*, and *Bacteroidota* (Fig. 1A). At the genus level, the top five genera were *Rothia*, *Streptococcus*, *Prevotella*, *Porphyromonas*, and *Actinomyces* (Fig. 1B). At the species level, *Rothia* species, *Streptococcus* species, *Actinomyces* species, and *Prevotella* species were commonly found (Fig. 1C). Among them, the top 10 species were *Rothia mucilaginosa*, *Streptococcus parasanguinis*, *Porphyromonas somerae*, *Prevotella melaninogenica*, *Streptococcus salivarius*, *Streptococcus mitis*, *Streptococcus_oralis*, *Neisseria_subflava*, *Actinomyces graevenitzii*, and *Prevotella pallens*. The distribution characteristics of oral bacteria in our studies were partly similar to those identified in other studies (15, 16). In conclusion, we elucidate the microflora distribution characteristics of lung cancer patients treated with immunotherapy.

To compare differences in bacteria diversity associated with immunotherapy sensitivity and resistance, the alpha and beta diversity of the saliva microbiome between the N and NR groups was compared. At the species level, the ACE index (Fig. S1A) and Chao1 index (Fig. S1B) were similar between the R and NR groups. The Simpson index (Fig. S1C) and Shannon index (Fig. S1D) showed a lower diversity trend in the R group, but the results did not reach statistical significance. For beta diversity analysis, PCOA based on Bray-Curtis distance (Fig. S1E) and Jaccard distance (Fig. S1F) at the species level were performed to visualize the dissimilarity of saliva taxonomy community between the two groups. The results showed that the two groups shared common species with similar abundance. There was no significant disparity in the diversity of oral bacteria between immunotherapy R and NR among NSCLC patients.

### Differential species associated with immunotherapy response

**Fig 2:**
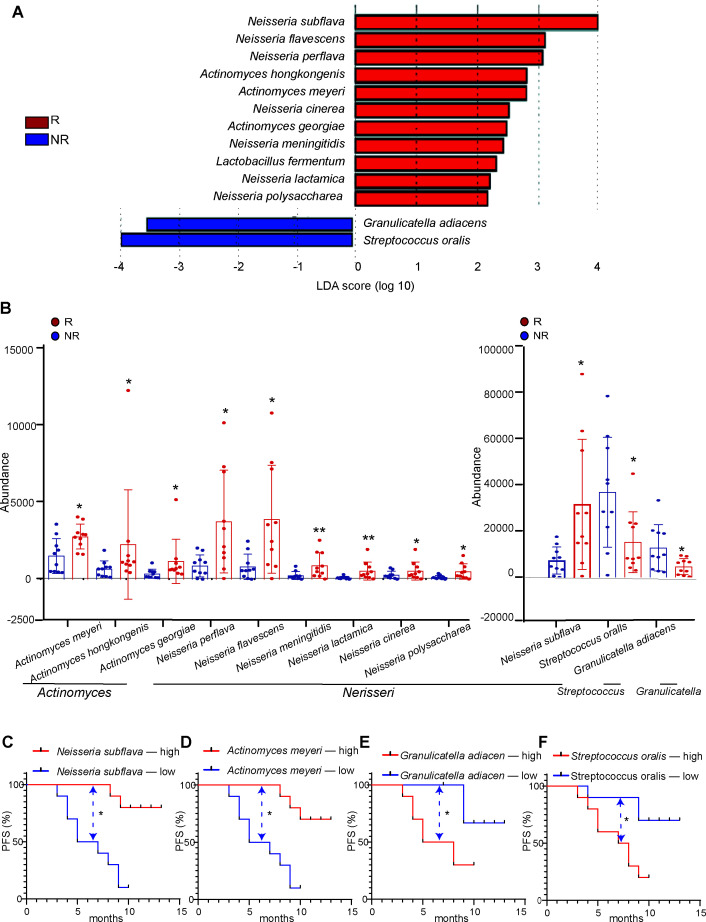
Differential bacterial species between NR and R patients. (**A**) Lefse analysis identified 13 differential species. Among them, 11 species were more enriched in the R group, and 2 species were more enriched in the NR group. N, responder; NR, non-responder. (**B**) Distribution and abundance of the main 12 differential bacterial species identified by LEfSe. *P* value was calculated via Mann-Whitney *U* test. *P < 0.05, **P < 0.01. N: responder; NR: non-responder. (**C–F**) Kaplan-Meier analysis of the PFS in lung cancer patients stratified by median abundance of each dominant differential species. High level *Neisseria subflava* (**C**) and *Actinomyces meyeri* (**D**) group has considerable survival advantage over low-level group. Low level *Granulicatella adiacens* (**E**) and *Streptococcus oralis* (**F**) group has considerable survival advantage over high-level group. *P* value was calculated via log-rank test. *P < 0.05. OS, overall survival; PFS, progression-free survival.

To further explore the specific species of oral bacteria related to immunotherapy efficacy, LEfSe analysis was performed between the NR and R groups. In total, 11 enriched species were identified in the R group, and 2 enriched species in the NR group. Among them, *Neisseria* species and *Actinomyces* species were commonly found (Fig. 2A). Specifically, *Neisseria subflava*, *Neisseria perflava*, *Neisseria flavescens*, *Neisseria meningitidis*, *Neisseria lactamica*, *Neisseria cinerea*, *Neisseria polysaccharea*, *Actinomyces meyeri*, *Actinomyces hongkongensis*, *Actinomyces georgiae*, and *Lactobacillus fermentum* were significantly increased in the R group, while *Granulicatella adiacens* and *Streptococcus oralis* were significantly increased in the NR group (Fig. 2A). Apart from *L. fermentum*, the other 12 differential species had an abundance ≥1,000 and were used for further analysis. The distribution and abundance of the 12 differential species are shown (Fig. 2B). Through Mann-Whitney *U* test, significant disparities in species distribution among these two groups were further revealed. *N. subflava* was most abundant among *Neisseria* species, while *A. meyeri* was most abundant among *Actinomyces* species in the R group. The two enriched species*—G. adiacens* and *S. oralis*—were with most abundance in the NR group. These 12 differential species were listed (Table S2). Our findings indicate that a high concentration of *Neisseria* and *Actinomyces* species is characteristic of immunotherapy responders in lung cancer. Conversely, a high enrichment of *G. adiacens* and *S. oralis* is characteristic of immunotherapy NRs in lung cancer.

Given the potential variability in the abundance of the saliva microbiota over time, our aim was to ascertain the stability of these 12 differential bacteria during lung cancer immunotherapy treatment. Saliva samples from all NR patients at the time of progression disease (PD) were collected again and subjected to mNGS sequencing. The results indicated that the abundance of these 12 differential species remained stable (Fig. S2), indicating its potential use as a predictor for immunotherapy efficacy. Spearman correlation analysis was performed to measure the relationship between the 12 differential species (Fig. S3). The result revealed a strong correlation among each of the seven *Neisseria* species and a similar strong correlation among each of the three *Actinomyces* species. Additionally, *G. adiacens* showed moderate association with *S. oralis*.

Furthermore, we test whether *N. subflava*, *A. meyeri*, *G. adiacens*, and *S. oralis* were associated with progression-free survival (PFS) in lung cancer immunotherapy. All patients were divided into two groups based on median levels of each species. Kaplan-Meier analysis showed that patients with higher abundance of *N. subflava* (Fig. 2C) and *A. meyeri* (Fig. 2D) tended to have longer PFS. Conversely, patients with higher abundance of *G. adiacens* (Fig. 2E) and *S. oralis* (Fig. 2F) tended to have shorter PFS. This indicates that the specific bacteria distribution is closely linked to treatment outcome of patients, further suggesting that the detection of bacteria can serve as a prognostic indicator of immunotherapy.

### Differential species associated with PD-L1 expression

**Fig 3:**
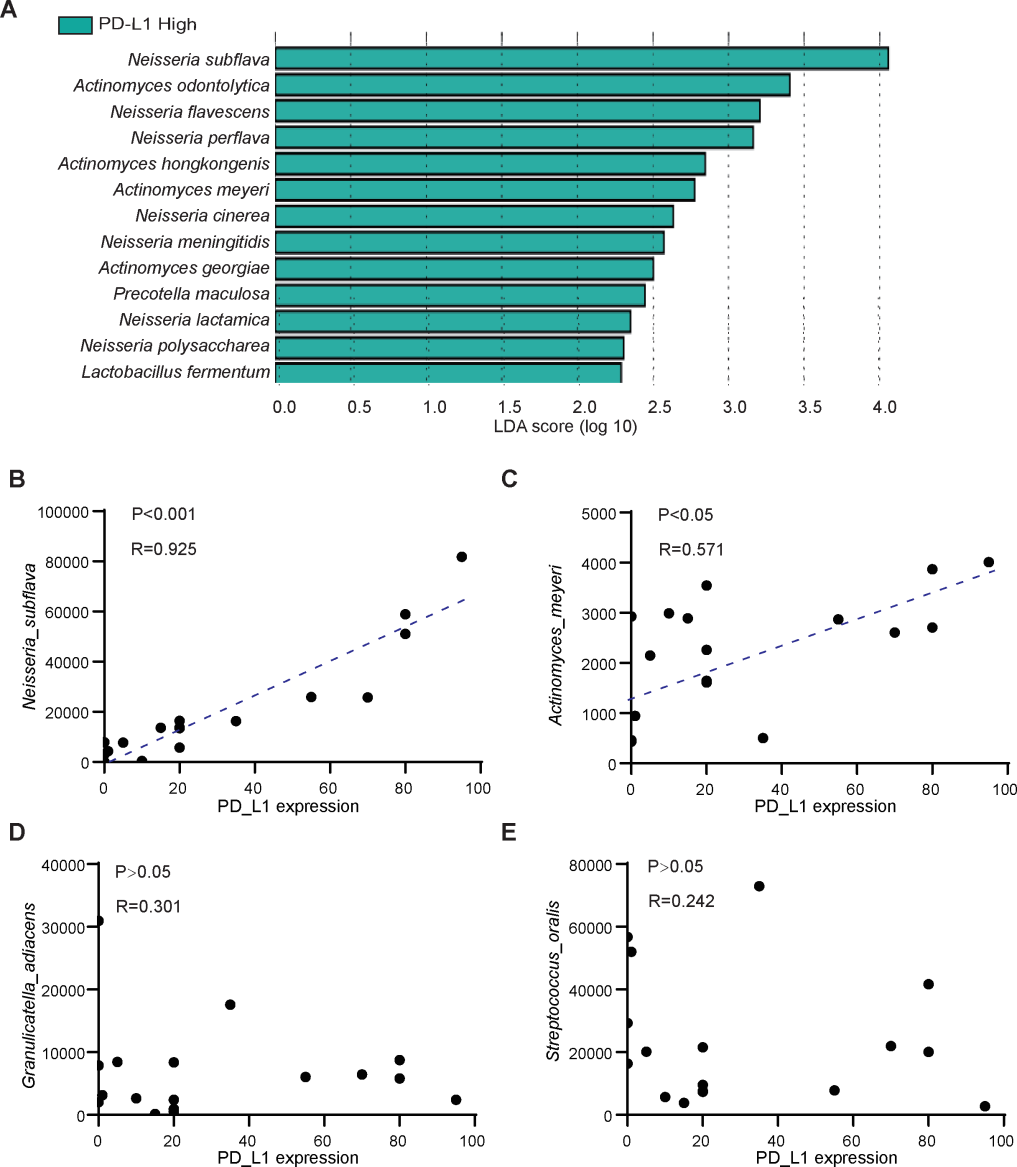
Relationship between saliva microbiome and PD-L1 expression. (**A**) Lefse analysis identified several differential bacterial species among patients with PD-L1 ≥10%. (**B–E**) Spearman correlation analysis between main differential bacterial species and PD-L1 expression. *Neisseria subflava* (**B**) and *Actinomyces meyeri* (**C**) were positively associated with PD-L1 expression. *Granulicatella adiacens* (**D**) and *Streptococcus oralis* (**E**) were not significantly associated with PD-L1 expression.

PD-L1 is an important therapeutic target and detection marker for immunotherapy. To further support the close connection between oral bacterial distribution and immunotherapy, we analyzed the expression and distribution between PD-L1 and different main bacterial of N and NR groups in lung cancer. Patients with PD-L1 <10% and PD-L1 ≥10% were divided into two groups. Interestingly, LEfSe analysis showed that most R enriched species, including *N. subflava*, *N. perflava*, *N. flavescens*, *N. meningitidis*, *N. lactamica*, *N. cinerea*, *N. polysaccharea, A. meyeri*, *A. hongkongensis* and *A. georgiae*, were also significantly increased in the PD-L1 high group (Fig. 3A). Spearman correlation analysis indicated that *N. subflava* was strongly correlated with PD-L1 expression (*P* < 0.001, *R* = 0.925) (Fig. 3B) and *A. meyeri* was moderately associated with PD-L1 expression (*P* < 0.05, *R* = 0.571) (Fig. 3C), while *G. adiacens* (Fig. 3D) and *S. oralis* (Fig. 3E) had no significant correlation with PD-L1 expression.

Consistent with our previous findings, in the R group, *Neisseria* and *Actinomyces* species are obviously enriched and have a positive relationship with PD-L1 expression, indicating that these bacteria are associated with immunotherapy sensitivity. On the contrary, *G. adiacens* and *S. oralis* were notably enriched in the NR group and associated with low PD-L1 expression, suggesting they are immunotherapy-resistant associated bacteria in lung cancer. These results emphasize the significance and importance of the different distribution of oral bacteria in lung cancer immunotherapy.

### Analysis of differences in metabolic patterns between NR and R groups

Nutrients serve as chemical signals that regulate cellular growth and metabolism. Overnutrition not only disrupts metabolic balance but also leads to excessive nutrient signaling, which in turn promotes abnormal cellular metabolism and proliferation, contributing to the development of various diseases, including cancer. Previous studies have shown that lung cancer immunotherapy resistance is closely related to abnormal nutritional signals (17). To investigate the metabolic pattern in people with different immunotherapy efficacy and find microbiome-related metabolites, LM/MS analysis was conducted. A total of 1,008 positive ion mode metabolites and 507 negative ion mode metabolites were identified in the saliva sample.

**Fig 4:**
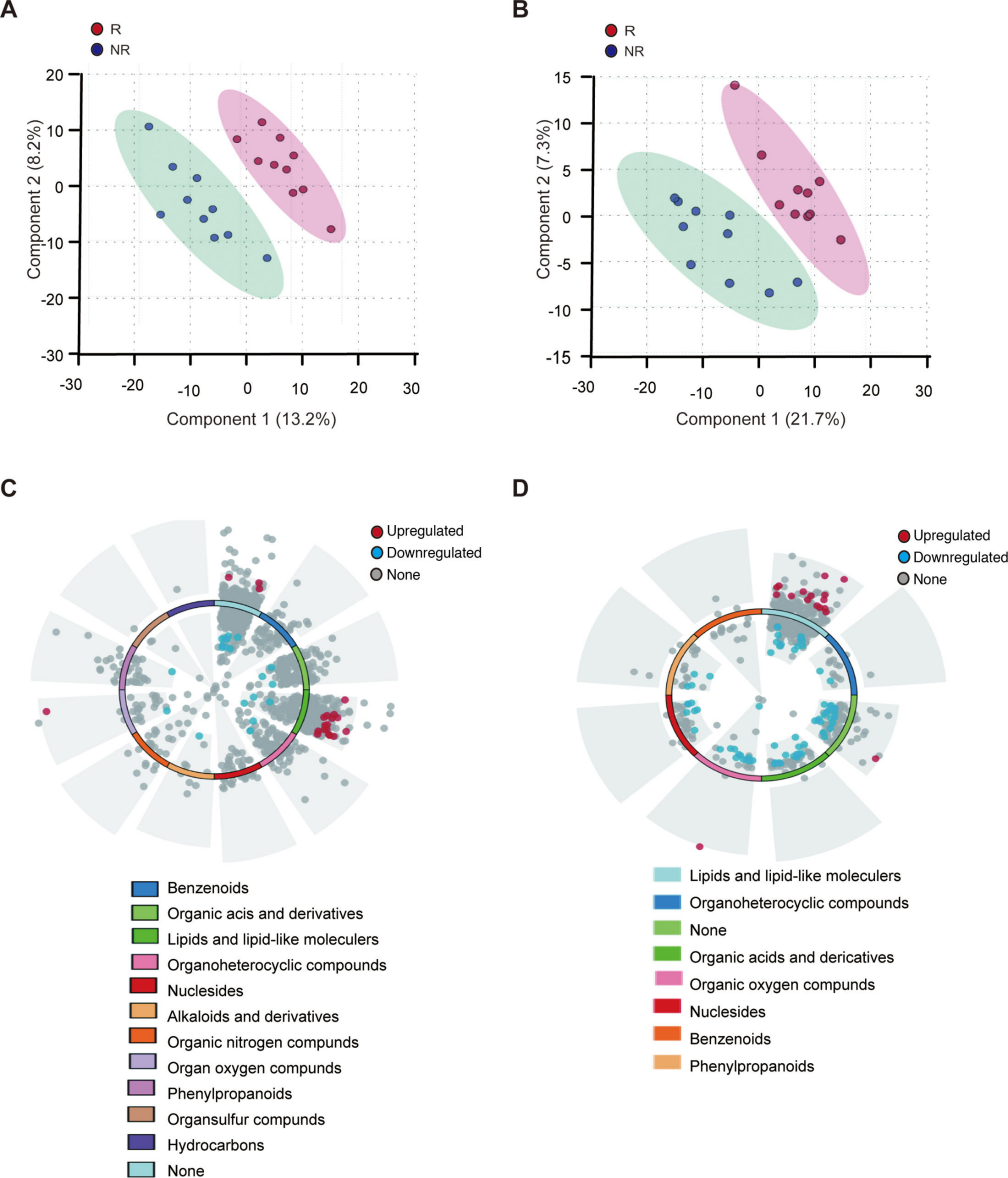
Metabolomics analysis revealed distinctive metabolic profile between NR and R groups. (**A and B**) PLS-DA analysis of positive ion model (**A**) and negative ion model (**B**) showed that metabolic patterns significantly differed between the two groups. N, responder; NR, non-responder. (**C and D**) Differential metabolites of positive ion model (**C**) and negative ion model (**D**) were shown in ring-shaped volcano plot. Most of them were lipids and lipid-like molecules.

Given the susceptibility of metabolomics to external factors and rapid changes, quality control (QC) samples are commonly used for ensuring data reliability. QC analysis of metabolites in both ion modes showed a high Pearson correlation coefficient (*R*^2^ > 0.9) (Fig. S4A and B), and the PLS-DA model clearly distinguished the metabolic profiles of NR and R (Fig. 4A and B). Differential metabolites between the NR and R groups were identified based on specific criteria: the VIP of the first two principal components of the PLS-DA model ≥1 ; fold change ≥ 1.5 or ≤ 0.67; *P* value < 0.05. The differential metabolites between the NR and R groups were listed in the ring-shaped volcano plot (Fig. 4C and D). A total of 84 potential biomarker metabolites were selected in negative ion mode, while a total of 40 potential biomarker metabolites were selected in positive ion mode.

**Fig 5:**
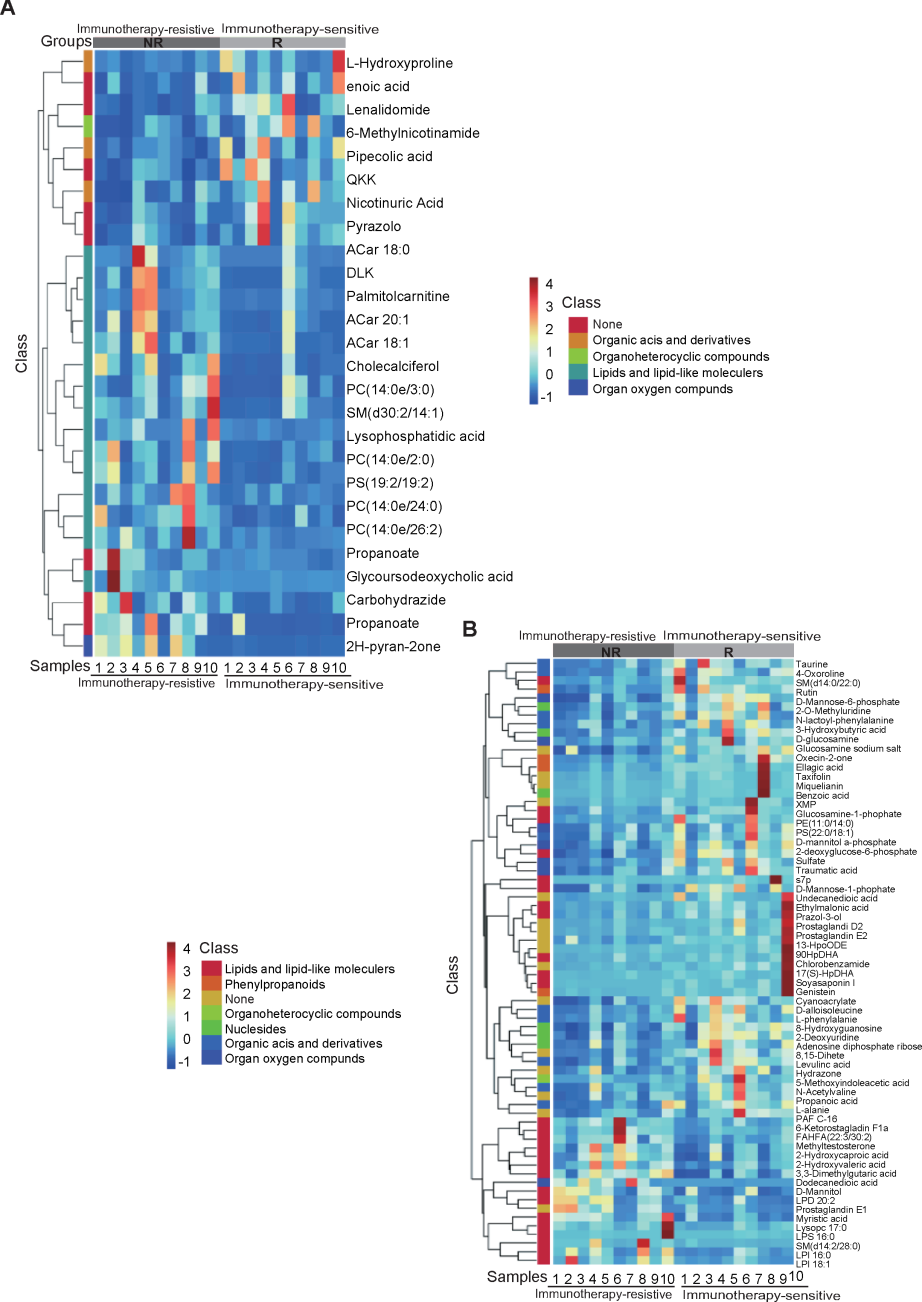
Lipid and lipid-like molecules were enriched in the NR group. (**A**) Hierarchical clustering heatmap for the differential metabolites identified through the positive ion mode of LC/MS analysis between the N and NR groups. Data are normalized based on *Z* score. N, responder; NR, non-responder. (**B**) Hierarchical clustering heatmap for the differential metabolites identified through the negative ion mode of LC/MS analysis between the N and NR groups. Data are normalized based on *Z* score. N responder; NR, non-responder. The gradient bar within the heatmaps, which denotes the relative abundance of metabolites, ranging from 4 to −1. The color red indicates an increase in the abundance of each metabolite in a given sample relative to the mean value, whereas blue signifies a decrease.

In order to ascertain whether these differential metabolites were stable during lung cancer treatment, saliva samples from all the NR patients at the time of PD were gathered again and proceeded with LM/MS analysis. The Wilcoxon rank test was used to compare differential metabolites at different time points. In total, 70 differential metabolites in the negative ion mode and 28 differential metabolites in the positive ion mode remained stable. These stable differential metabolites were listed in Table S3 and were used for further analysis. The majority of differential metabolites were lipids and lipid-like molecules in both negative ion mode (26 out of 70, 37%) and positive ion mode (14 out of 28, 50%). Among the differential lipids and lipid-like metabolites, most of them (15 in negative ion mode and 14 in positive ion mode) were upregulated in the NR group. The abundance heatmap of the stable differential metabolites in two ion modes was shown (Fig. 5A and B). Most of the differential lipids and lipid-like metabolites were clustered together. Interestingly, correlation analysis showed that the total abundance of all lipids and lipid-like metabolites was negatively associated with PD-L1 expression (Fig. S5) (*P* = 0.029, *R* = −0.534 for negative ion mode and *P* = 0.018, *R* = −0.572 for positive ion mode). These results revealed abnormal fat metabolism was highly enriched in the NR group and was closely related to the low expression of PD-L1. In conclusion, we elucidate the metabolic characteristics of lung cancer patients treated with immunotherapy.

### Specific correlation between different saliva microbiota species and different fat metabolites

**Fig 6:**
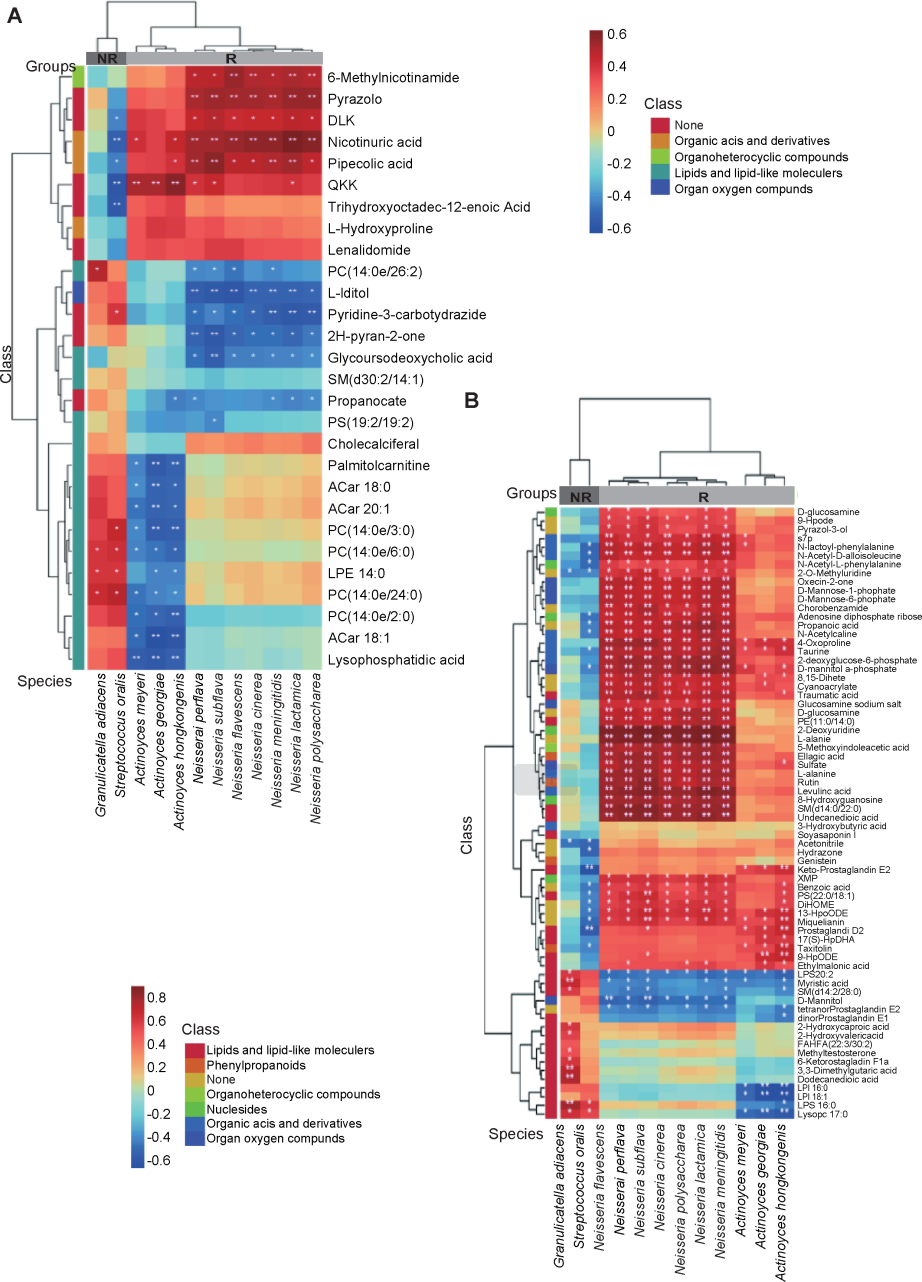
Several differential metabolites, including lipids and lipid-like metabolites, were significantly associated with some bacteria species. (**A**) Correlation heatmap for the differential metabolites identified through the positive ion mode of LC/MS analysis and the differential species between the N and NR groups. Spearman correlation was conducted and color intensity indicated *r* value. N, responder; NR, non-responder. (**B**) Correlation heatmap for the differential metabolites identified through the negative ion mode of LC/MS analysis and the differential species between the N and NR groups. Spearman correlation was conducted and color intensity indicated *r* value. N, responder; NR, non-responder. The gradient bar within the heatmap represents the range of Spearman correlation coefficients, extending from −0.6 to 0.8. Red coloration corresponds to a positive correlation, while blue indicates a negative correlation. The intensity of the coloration is directly proportional to the strength of the correlation; deeper shades imply a stronger correlation. *P value<0.05; **P value<0.01.

To further reveal the specific correlation between differential microbiota and metabolites, spearman correlation analysis was conducted (Fig. 6A and B). Several lipids and lipid-like metabolites were associated with differential microbiota species. Precisely, most differential phosphocholine and acylcarnitines in positive ion mode were negatively associated with differential *Actinomyces* species. Most phosphocholine metabolites were positively associated with *G. adiacens* or *S. oralis*. PC (14:0e/26:2) and glycoursodeoxycholic acid were negatively associated with differential *Neisseria* species. In terms of differential lipids and lipid-like metabolites in negative ion mode, most NR group enriched lipids and lipid-like metabolites were positively associated with *G. adiacens*. All deferential monoacyl glycerophosphoinositols were negatively associated with differential *Actinomyces* species. LPS 20:2, myristic acid, SM (d14:0/22:0) and tetranor Prostaglandin E2 were negatively associated with differential *Neisseria* species, while most R group enriched lipids and lipid-like metabolites were positively associated with differential *Neisseria* species (Fig. 6A and B). In summary, certain lipids and lipid-like metabolites, positively enriched in the NR group, had a significant connection with specific saliva species associated with immunotherapy efficacy. This suggests that the specific oral microbiota distribution associated with lung cancer immunotherapy was closely related to abnormal regulation of fat metabolism signals.

### The sensitivity of immunotherapy is correlated with abnormal fat metabolism in human lung cancer samples

**Fig 7:**
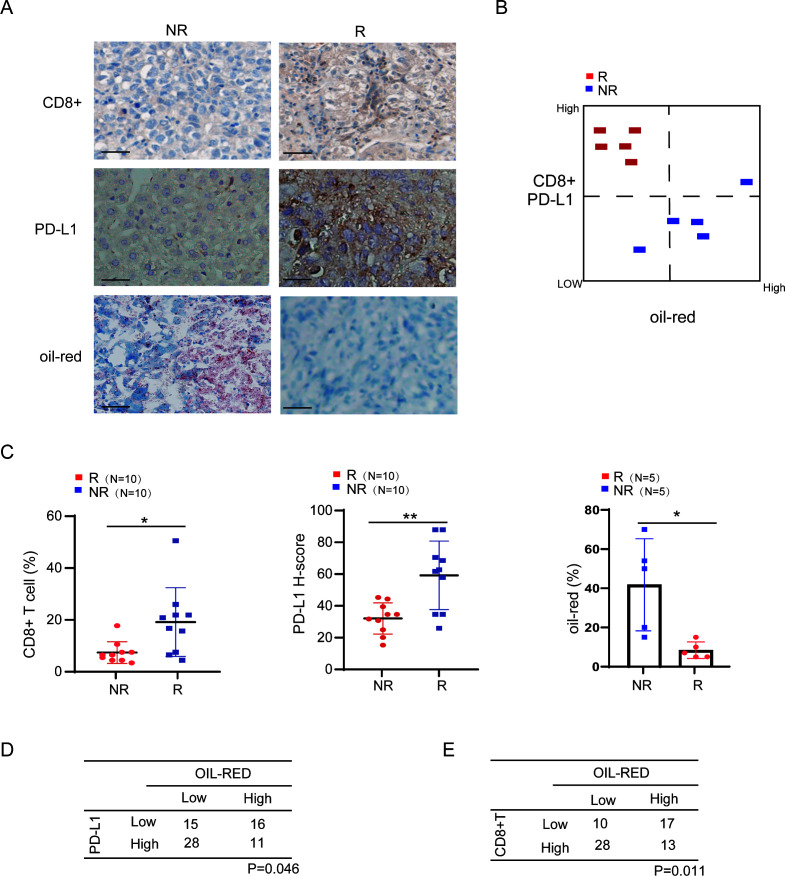
The sensitivity of immunotherapy is correlated with abnormal lung fat metabolism in human clinical samples. (**A**) The fat metabolism level is closely correlated with immunotherapy sensitivity in lung cancer. Representative IHC staining image of lung cancer tissues treated with immunotherapy with CD8, PD-L1, and oil-red stain (scale bar = 50 µm). N, responder; NR, non-responder. (**B**) Faulty fat metabolism is elevated in immunotherapy-resistance lung cancer tissue. Resistive to immunotherapy, human lung tissue sections marked by low CD8 and PD-L1 have high oil-red expression, and vice versa. N, responder; NR, non-responder. (C) Fat metabolism, CD8, and PD-L1 expression were indicated by scatter plots in lung cancer treated with immunotherapy. NR group showed low expression of CD8 and PD-L1, and high oil-red stain compared with the R group. *P < 0.05, **P < 0.01. N, responder; NR, non-responder. (**D and E**) The IHC score of oil-red stain is negatively correlated with CD8 or PD-L1 expression in lung cancer treated with immunotherapy. Seventy samples collected from lung cancer patients receiving immunotherapy were indicated CD8, PD-L1, and oil-red stain by IHC experiments.

Based on the aforementioned sequencing analysis results, we conclude that the abnormal fat metabolism associated with dysbacteriosis is closely related to the sensitivity of lung cancer immunotherapy. To further explore the clinical significance of abnormal fat metabolism in lung cancer immunotherapy, we asked whether it occurs in human lungs. To this end, we performed oil-red staining and immunohistochemistry (IHC) staining with anti-CD8 and anti-PD-L1 antibody on human lung cancer tissue samples received immunotherapy. Through IHC assay, by macroscopic observation, we found that oil-red staining was generally elevated in immunotherapy-resistive lung cancer tissues compared to that in immunotherapy-sensitive lung cancer tissues, and accompanied by low CD8 and PD-L1 expression on lung carcinoma cells (Fig. 7A and B). When quantifying the expression of immune checkpoint CD8, PD-L1, and the level of abnormal fat metabolism in lung cancer tissues received immunotherapy, we further proved that immunoactivation markers CD8 and PD-L1 were significantly higher in the R group. Interestingly, fat metabolism was obviously suppressed in the R group, compared with NR (Fig. 7C). We also performed oil-red staining with a tissue microarray consisting of 70 lung cancer samples from 39 patients with high PD-L1 and 31 patients with low PD-L1 expression. Through chi-square test analysis, a negative correlation between oil-red stain and PD-L1 was observed (Fig. 7D), implying that fat metabolism pathway regulates PD-L1-associated immune signals in human lung cancer. We also conducted IHC for CD8 and analyzed the relationship between immune cells and oil-red stain. Similar results were also shown (Fig. 7E). Therefore, we proved that abnormal fat metabolism is closely related to the sensitivity of lung cancer immunotherapy.

### Abnormal fat metabolism regulates T cell activity and infiltration through PD-L1

**Fig 8:**
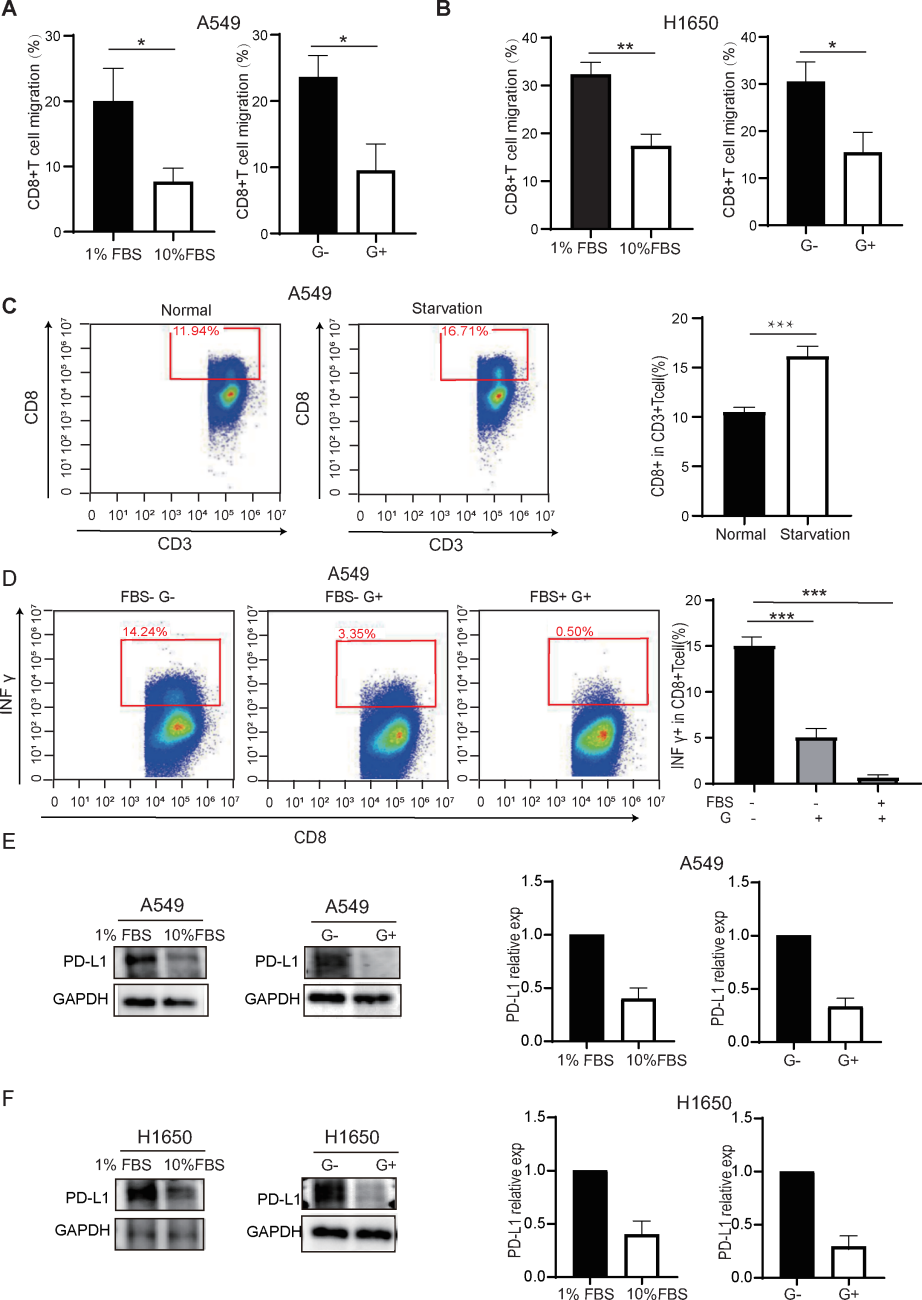
Abnormal metabolism reduces the number and activity of CD8^+^ T cells through PD-L1 in lung carcinoma cells. (**A and B**) Glucose (G) and fetal bovine serum (FBS) starvation activated the migration rate of CD8^+^ T cells in lung cancer. A549 and H1650 cells were deprived from FBS or G for 24 h, and then co-cultured with human T cells for 12 h. The cell migration rate of CD8^+^ T cells was detected by T cell migration experiment. Data (mean ± SD，*n* = 3). *<0.05, **<0.01. G− : without glucose, G+: with glucose, 1% FBS: with 1% serum, and 10% FBS: with 10% serum. G and FBS starvation activated T cell infiltration migration rate in lung cancer. A549 cells were deprived from FBS and G for 24 h, and then co-cultured with human T cells for 36 h. The secretion of CD8 in CD3^+^ T cells was detected by flow cytometry experiment. Data (mean ± SD，*n* = 3). ***<0.001. Normal: full medium with FBS and G, Starvation: without FBS and G. (**C**) G and FBS starvation activated IFN γ^+^CD8^+^ T cell in lung cancer. A549 cells were deprived from FBS, G, and FBS + G for 24 h, and then co-cultured with T cells for 36 h. The secretion of INF γ in CD8^+^ T cells was detected by flow cytometry experiment. Data (mean ± SD，*n* = 3). ***<0.001. G− : without glucose, G+: with glucose, FBS−: without serum, and FBS+: with serum. (**E and F**) Abnormal lipid metabolism activated the expression of PD-L1 in lung carcinoma cells. A549 and H1650 cells were deprived from FBS (serum) and G (glucose) for 24 h, and then the expression level of immune checkpoint PD-L1 was tested by Western blot experiments. GAPDH was used as a loading control. Data (mean ± SD，*n* = 3). G−: without glucose, G+: with glucose, 1% FBS: with 1% serum, and 10% FBS: with 10% serum.

Immune cells play a crucial role in response to immunotherapy. We asked if T cells respond to fat signal stimulation in lung carcinoma cells. To this end, T cell migration experiments were used to measure CD8^+^ T cell activity. Since fat intervention in cell culture is difficult to achieve and nutrient signals can regulate and transform each other, we used fetal bovine serum (FBS) and glucose (G) starvation to partially mimic fat starvation *in vitro* experiment. When lung cancer cells with different nutritional starvation states were co-cultured with human CD8^+^ T cells for 12 h, the migration activity of CD8^+^ T cells was notably activated under starvation from FBS and G in A549 and H1650 cells (Fig. 8A and B). Similarly, FBS and G starvation in A549 and H1650 cells resulted in upregulation of the differentiation of CD3^+^ T cells into CD3^+^CD8^+^ T cells (Fig. 8C). Furthermore, starvation also increased the secretion of IFN gamma, thereby activating CD8^+^ T cells (Fig. 8D). These results indicate that the activity and infiltration of CD8^+^ T cells affected by G and FBS signals, consistent with our previous observations. The sensitivity of immunotherapy is correlated with abnormal fat metabolism. Moreover, we found that FBS and G starvation increased immune checkpoint PD-L1 expression (Fig. 8E and F), suggesting that PD-L1 might be a mediator of metabolism signals to T cells.

## DISCUSSION

The oral cavity supports a diverse microbiome interacting intricately with the host’s immune system. Dysbiosis of the oral microbiota can lead to various diseases (18), and in lung cancer, it modulates processes like apoptosis inhibition, proliferation stimulation, and chronic inflammation induction (19, 20), with specific bacteria like *P. gingivalis* and *Fusobacterium nucleatum* linked to progression and poor prognosis (21–23). Saliva, a rich biological fluid secreted by oral glands, contains a complex milieu of microorganisms, metabolites, and proteins. Saliva contains microorganisms, metabolites, and proteins. As a non-invasive diagnostic tool with potential similar to blood in cancer prognosis and therapy monitoring, it reflects systemic conditions and needs further exploration (24, 25).

In this investigation, we scrutinized the salivary microbiota and metabolomic profiles of Chinese patients with advanced NSCLC stratified by immunotherapy responsiveness. The results revealed a significantly enriched microbial community in R versus NR, alongside distinct metabolic signatures. Correlation analyses underscored a strong association between differential bacterial species and fat metabolites. Experiments data further confirmed the relationship between lung cancer immunotherapy resistance and abnormal metabolites while also exploring the regulation of CD8^+^ T cells and PD-L1 under fat metabolism in lung cancer immunotherapy. These findings highlight the pivotal role of salivary microbiota imbalance leading to metabolic dysregulation as a critical determinant of varied immunotherapy responses in NSCLC, thus facilitating patient selection for immunotherapy.

*Neisseria*, a common oral microbiota constituent, was reported to be related to lung cancer prognosis (26, 27). In our study, we found that higher *Neisseria* species abundance correlated with a better immunotherapy response. Further analysis revealed that in immunotherapy-sensitive patients, *N. subflava*, *N. perflava*, *N. flavescens*, *N. meningitidis*, *N. lactamica*, *N. cinerea*, and *N. polysaccharea*, were mainly elevated. Among them, *N. subflava* was most prevalent and was associated with longer PFS and higher PD-L1 expression. A recent study also found that the abundance of *N. perflava* in alveolar lavage fluid was associated with immunotherapy response in NSCLC patients (28). These findings suggested that the detection and intervention of *Neisseria* species play a role in prognosis prediction and efficacy promotion in lung cancer immunotherapy. Several *Neisseria* species are thought to have roles in maintaining immune ignorance in the acquired immune response (29). Powell et al. studied the stimulation of macrophages with *Neisseria* species in the oral microbiota of mice and reported that macrophages stimulated interleukin 6 (IL-6) (30). Interestingly, multiple studies suggest IL-6 is associated with increased PD-L1 expression through various signaling pathways, promoting immune evasion in multiple cancer types (31–33). This suggests a possible mechanism where *Neisseria* species, through their interaction with macrophages, could indirectly influence the expression of PD-L1.

Saliva *Actinomyces* species, including *A. meyeri*, *A. hongkongensis*, and *A. georgiae*, were other common species positively associated with immunotherapy response and PD-L1 expression. *Actinomyces* species are frequently present in patients with various solid tumors (34–36). The genus *Actinomyces* was associated with pathology type (37) and the advanced stage of lung cancer (38). Furthermore, it was observed that *Actinomyces* species can activate human monocyte-derived dendritic cells (39). Several studies have suggested that circulating dendritic cells correlate with PD-L1 expression, impacting tumor growth, T cell responses, and immunotherapy efficacy in various cancers (40, 41).

In our study on NSCLC patients, we found that a higher abundance of *G. adiacens* and *S. oralis* were associated with poor immunotherapy efficacy. *G. adiacens* was more abundant in lung cancer patients and may be a biomarker (42, 43). Its biofilms significantly induce the production of pro-inflammatory cytokines, such as IL-1β, IL-8, and TNF-α, from peripheral blood mononuclear cells (44). In lung cancer, IL-1β is a key driver of inflammation in the tumor microenvironment, and its inhibition may enhance the efficacy of PD-1 inhibitors (45). Elevated serum IL-8 levels are associated with poor prognosis and reduced efficacy of ICIs, including anti-PD-1 and anti-CTLA-4 therapies (46). *S. oralis*, another NR group enriched species, is enriched in the lower airways of lung cancer patients. The enrichment of *S. oralis* upregulated phosphoinositide 3-kinase (PI3K) signaling pathway (47), thus may hamper the efficacy of immunotherapy.

Dysregulated lipid metabolism, a cancer hallmark, affects tumor cell behaviors (48, 49). In our cohort, non-targeted metabolomics sequencing revealed that lipids and lipid-like metabolites were the most common differential metabolites associated with immunotherapy efficacy and were associated with PD-L1 expression. Importantly, most of them were associated with the differential species. Aberrant lipid metabolism in the tumor microenvironment plays a significant role in suppressing immune responses and affects various immune cells, including T cells and macrophages (50). Elevated lipid levels in the tumor micro-environment are linked to immune dysfunction, particularly in CD8^+^ tumor-infiltrating lymphocytes (TILs). The accumulation of oxidized lipids via the CD36 receptor leads to T cell dysfunction, which can be mitigated by inhibiting lipid peroxidation pathways (51). Consistent with these, our *in vitro* experimental data also show that interfering with lipid metabolism of lung cancer cells can obviously increase CD8^+^ T cell activation and migration, and upregulated the expression of PD-L1. Besides, IHC staining of tissue samples from lung cancer patients who had received immunotherapy also confirmed that the response to immunotherapy was closely related to the level of lipid accumulation.

In summation, through the integration of oral mNGS and LC/MS analysis, we comprehensively profile saliva microbiota and metabolic signatures in patients with NSCLC. Notably, the presence of specific species such as *Neisseria* and *Actinomyces* species provided distinctive biomarkers that differentiated NR from R and were linked to PD-L1 expression. Furthermore, perturbed lipid metabolism emerged as a salient characteristic among NR patients. The remarkable correlations observed between specific saliva microbiota, lipid metabolites, and immunotherapy efficacy highlight their potential as important factors influencing treatment outcomes for NSCLC patients.

However, it is important to acknowledge that the current study has several limitations. First, the relatively small sample size may limit the generalizability of the microbial composition and metabolite-based diagnostic models. Therefore, it is crucial to externally validate our findings through larger, multicenter studies. Additionally, accurately assessing saliva metabolites and distinguishing them from host-derived metabolites versus microbial-derived metabolites remains a challenging task. Furthermore, the study adopts a cross-sectional design, which only captures the microbiome-metabolome associations at a single time point. Further investigation is necessary to understand the underlying mechanisms of microbiota involvement and causal relationships.

## Data Availability

Data supporting the findings of this work are available within the paper and in the supplemental files. The original data of mNGS and metabolomics have been uploaded in FigShare (https://figshare.com/articles/dataset/dx_doi_org_10_6084_m9_figshare_6025748/6025748).

## SUPPLEMENTAL MATERIAL

Supplemental material msystems.01115-24-s0001.docx Supplemental tables, figures, and materials and methods.

Source data msystems.01115-24-s0002.xlsx Source data of all main figures.

ASM does not own the copyrights to Supplemental Material that may be linked to, or accessed through, an article. The authors have granted ASM a non-exclusive, world-wide license to publish the Supplemental Material files. Please contact the corresponding author directly for reuse.
