# Supplementary material for: Variations in salivary microbiome and metabolites are associated with immunotherapy efficacy in patients with advanced NSCLC
Source: mSystems. 2025 Feb 10;10(3):e01115-24. doi: 10.1128/msystems.01115-24 (PMC11915794; doi:10.1128/msystems.01115-24)
Supplement: Supplemental material — Supplemental tables, figures, and materials and methods. [file msystems.01115-24-s0001.docx]

**Supporting Information**

**Variations in salivary microbiome and metabolites are associated with immunotherapy efficacy in patients with advanced NSCLC**

Huang et al.

1. **Supplementary Table S1-S3**
2. **Supplementary Figure S1-S5**

**3. Supplementary Materials and Methods**

1. **Supplementary Tables**

**Table S1** **Demographics and clinical characteristics of advanced NSCLC patients**

| Parameter | NR(10) | R(10) | P-value |
| --- | --- | --- | --- |
| Age,y,(mean±SD） | 59.80±6.29 | 52.9±18.05 | 0.277^a^ |
| BMI(mean±SD） | 22.08±2.76 | 20.06±3.77 | 0.333^a^ |
| Sex |  |  | 1.000^b^ |
| Male | 10 (100%) | 9 (90%) |  |
| Female | 0 (0%) | 1 (10%) |  |
| Smoking status |  |  | 0.370^b^ |
| Smoker | 9 (90%) | 6 (60%) |  |
| Nonsmoker | 1 (10%) | 4 (40%) |  |
| Pathological type |  |  | 0.582^b^ |
| Adenocarcinoma | 7 (70%) | 9 (90%) |  |
| squamous carcinoma | 3 (30%) | 1 (10%) |  |
| clinical stages |  |  | 1.000^b^ |
| Stage III | 3 (30%) | 3 (30%) |  |
| Stage IV | 7 (70%) | 7 (70%) |  |
| PD-L1 expression |  |  | 0.0026^b^ |
| PD-L1≥10% | 2 (20%) | 9 (90%) |  |
| PD-L1＜10% | 8 (80%) | 1 (90%) |  |

BMI body mass index, NR non responder, R responder. an independent t-test, b Fisher’s exact test.

**Table S2 Differential species among NR and R group**

| Species | Mean Abundance（NR） | Mean Abundance  (R) | LDA score | P-value |
| --- | --- | --- | --- | --- |
| *Neisseria_subflava* | 7562.64 | 30752.474 | 3.96 | 0.008^a^ |
| *Neisseria_perflava* | 977.60 | 3889.804 | 3.06 | 0.013^a^ |
| *Neisseria_flavescens* | 951.17 | 4023.372 | 3.10 | 0.016^a^ |
| *Neisseria_meningitidis* | 261.72 | 928.441 | 2.42 | 0.023^a^ |
| *Actinomyces_meyeri* | 1714.63 | 2620.344 | 2.79 | 0.028^a^ |
| *Neisseria_lactamica* | 147.50 | 567.081 | 2.20 | 0.028^a^ |
| *Neisseria_cinerea* | 306.61 | 1117.884 | 2.51 | 0.034^a^ |
| *Neisseria_polysaccharea* | 139.36 | 527.855 | 2.16 | 0.041^a^ |
| *Actinomyces_hongkongensis* | 818.49 | 2314.780 | 2.80 | 0.041^a^ |
| *Actinomyces_georgiae* | 506.14 | 1162.273 | 2.48 | 0.049^a^ |
| *Streptococcus_oralis* | 29954.22 | 14749.977 | 3.99 | 0.049^a^ |
| *Granulicatella_adiacens* | 9481.12 | 4577.990 | 3.55 | 0.041^a^ |

a: Mann-Whitney U test

**Table S3 Differential metabolites among NR and R group**

| Metabolites | Class I | | Fold change | | P-value | VIP score | |
| --- | --- | --- | --- | --- | --- | --- | --- |
| Positive ion mode | | | | | | | |
| (12Z)-9,10,11-trihydroxyoctadec-12-enoic acid |  | | 0.42 | | 0.0347 | 1.60 | |
| 3,5-dimethyl-1-phenyl-1,5-dihydro-4H-pyrazolo[4,3-c]quinolin-4-one |  | | 0.45 | | 0.0310 | 1.91 | |
| QKK |  | | 0.47 | | 0.0142 | 1.54 | |
| 4-methoxy-6-[2-(4-methoxyphenyl)ethyl]-2H-pyran-2-one |  | | 3.82 | | 0.0035 | 2.27 | |
| L-Iditol | Organic oxygen compounds | | 39.23 | | 0.0004 | 2.56 | |
| Cholecalciferol | Lipids and lipid-like molecules | | 3.10 | | 0.0323 | 1.83 | |
| Nicotinuric Acid | Organic acids and derivatives | | 0.49 | | 0.0267 | 1.66 | |
| PS (19:2/19:2) | Lipids and lipid-like molecules | | 3.91 | | 0.0208 | 2.02 | |
| ACar 18:1 | Lipids and lipid-like molecules | | 2.83 | | 0.0405 | 1.49 | |
| Pipecolic acid | Organic acids and derivatives | | 0.45 | | 0.0158 | 1.88 | |
| PC (14:0e/26:2) | Lipids and lipid-like molecules | | 4.84 | | 0.0101 | 2.11 | |
| Lenalidomide |  | | 0.32 | | 0.0139 | 1.49 | |
| ACar 20:1 | Lipids and lipid-like molecules | | 3.73 | | 0.0408 | 1.65 | |
| PC (14:0e/2:0) | Lipids and lipid-like molecules | | 5.41 | | 0.0095 | 2.14 | |
| PC (14:0e/24:0) | Lipids and lipid-like molecules | | 2.50 | | 0.0079 | 1.94 | |
| PC (14:1e/6:0) | Lipids and lipid-like molecules | | 4.19 | | 0.0117 | 2.04 | |
| SM (d30:2/14:1) | Lipids and lipid-like molecules | | 4.43 | | 0.0474 | 1.72 | |
| Oleoyl-L-alpha-lysophosphatidic acid | Lipids and lipid-like molecules | | 3.25 | | 0.0154 | 1.99 | |
| ethyl 3-oxo-3-(1H-pyrazol-5-ylamino)propanoate |  | | 3.48 | | 0.0393 | 1.29 | |
| L-Hydroxyproline | Organic acids and derivatives | | 0.36 | | 0.0466 | 1.20 | |
| 6-Methylnicotinamide | Organoheterocyclic compounds | | 0.42 | | 0.0462 | 1.75 | |
| LPE 14:0 | Lipids and lipid-like molecules | | 2.41 | | 0.0498 | 1.72 | |
| Glycoursodeoxycholic acid | Lipids and lipid-like molecules | | 8.82 | | 0.0403 | 1.74 | |
| ACar 18:0 | Lipids and lipid-like molecules | | 3.45 | | 0.0344 | 1.67 | |
| DLK |  | | 0.49 | | 0.0321 | 1.51 | |
| PC (14:0e/3:0) | Lipids and lipid-like molecules | | 2.49 | | 0.0284 | 1.86 | |
| N'3-(3,4,5-trimethoxybenzylidene)pyridine-3-carbohydrazide |  | | 2.87 | | 0.0370 | 1.60 | |
| Palmitoylcarnitine | Lipids and lipid-like molecules | | 2.96 | | 0.0355 | 1.52 | |
| Negative ion mode | | | | | | | |
| 8,15-Dihete |  | 0.30 | | 0.000 | | | 1.96 |
| PS (22:0/18:1) | Lipids and lipid-like molecules | 0.17 | | 0.001 | | | 1.94 |
| ethyl 3-(1,3-benzodioxol-5-ylamino)-2-cyanoacrylate |  | 0.40 | | 0.001 | | | 1.61 |
| Taxifolin | Phenylpropanoids and polyketides | 0.32 | | 0.001 | | | 1.74 |
| UDP-N-acetyl-alpha-D-glucosamine | Nucleosides, nucleotides, and analogues | 0.29 | | 0.001 | | | 1.80 |
| D-(-)-Mannitol | Organic oxygen compounds | 24.36 | | 0.001 | | | 1.94 |
| Prostaglandin D2 | Lipids and lipid-like molecules | 0.23 | | 0.002 | | | 1.68 |
| Levulinic acid | Organic acids and derivatives | 0.48 | | 0.002 | | | 1.41 |
| Myristic Acid | Lipids and lipid-like molecules | 3.16 | | 0.002 | | | 1.73 |
| 3,8,9-trihydroxy-10-propyl-3,4,5,8,9,10-hexahydro-2H-oxecin-2-one |  | 0.47 | | 0.002 | | | 1.49 |
| D-Mannose 6-phosphate | Organic oxygen compounds | 0.38 | | 0.003 | | | 1.69 |
| Traumatic acid | Lipids and lipid-like molecules | 0.46 | | 0.003 | | | 1.31 |
| Dodecanedioic acid | Lipids and lipid-like molecules | 2.04 | | 0.004 | | | 1.41 |
| N1-[4-(cyanomethyl)phenyl]-4-chlorobenzamide |  | 0.21 | | 0.004 | | | 1.24 |
| Ellagic acid | Phenylpropanoids and polyketides | 0.33 | | 0.004 | | | 1.58 |
| 8-Hydroxyguanosine | Nucleosides, nucleotides, and analogues | 0.47 | | 0.005 | | | 1.49 |
| 2'-O-Methyluridine | Nucleosides, nucleotides, and analogues | 0.40 | | 0.005 | | | 1.49 |
| Rutin | Phenylpropanoids and polyketides | 0.40 | | 0.005 | | | 1.18 |
| Miquelianin |  | 0.27 | | 0.006 | | | 1.60 |
| Ethylmalonic acid | Lipids and lipid-like molecules | 0.36 | | 0.007 | | | 1.55 |
| Taurine | Organic acids and derivatives | 0.32 | | 0.007 | | | 1.56 |
| Boc-beta-cyano-L-alanine | Organic acids and derivatives | 0.42 | | 0.007 | | | 1.44 |
| 2-(2-pyridyl)-2-{2-[4-(trifluoromethoxy)phenyl]hydrazono}acetonitrile |  | 0.39 | | 0.008 | | | 1.16 |
| 13-Hpotre(R) |  | 0.20 | | 0.008 | | | 1.56 |
| N-Acetyl-D-galactosamine 4-sulfate | Organic oxygen compounds | 0.36 | | 0.008 | | | 1.47 |
| α-D-Mannose 1-phosphate | Organic oxygen compounds | 0.39 | | 0.009 | | | 1.41 |
| s7p | Organic oxygen compounds | 0.30 | | 0.010 | | | 1.37 |
| 6-Ketoprostaglandin F1α | Lipids and lipid-like molecules | 2.94 | | 0.010 | | | 1.37 |
| N-Acetyl-D-alloisoleucine | Organic acids and derivatives | 0.28 | | 0.012 | | | 1.47 |
| 2-Deoxyuridine | Nucleosides, nucleotides, and analogues | 0.48 | | 0.012 | | | 1.22 |
| SM (d14:0/22:0) | Lipids and lipid-like molecules | 0.36 | | 0.013 | | | 1.35 |
| SM (d14:2/28:0) | Lipids and lipid-like molecules | 8.80 | | 0.013 | | | 1.47 |
| 2-[(3-methylbenzo[b]thiophen-2-yl)carbonyl]benzoic acid |  | 0.21 | | 0.014 | | | 1.25 |
| 13,14-dihydro-15-keto Prostaglandin E2 | Lipids and lipid-like molecules | 0.27 | | 0.014 | | | 1.39 |
| 13,14-dihydro-15-keto-tetranor Prostaglandin E2 |  | 5.19 | | 0.014 | | | 1.29 |
| LPS 20:0 | Lipids and lipid-like molecules | 2.12 | | 0.015 | | | 1.22 |
| Methyltestosterone | Lipids and lipid-like molecules | 2.17 | | 0.015 | | | 1.31 |
| 4-Oxoproline | Organic acids and derivatives | 0.45 | | 0.016 | | | 1.22 |
| Adenosine diphosphate ribose | Nucleosides, nucleotides, and analogues | 0.38 | | 0.016 | | | 1.46 |
| N-Sulfo-glucosamine sodium salt | Organic oxygen compounds | 0.39 | | 0.017 | | | 1.36 |
| LPI 18:1 | Lipids and lipid-like molecules | 3.92 | | 0.018 | | | 1.40 |
| Undecanedioic acid | Lipids and lipid-like molecules | 0.14 | | 0.019 | | | 1.39 |
| PAF C-16 |  | 0.48 | | 0.019 | | | 1.25 |
| XMP | Nucleosides, nucleotides, and analogues | 0.24 | | 0.019 | | | 1.38 |
| 4-chlorobenzaldehyde 1-[4-(1,3-oxazol-5-yl)phenyl]hydrazone |  | 0.50 | | 0.021 | | | 1.03 |
| 3-Hydroxybutyric acid | Organic acids and derivatives | 0.44 | | 0.023 | | | 1.17 |
| Soyasaponin I | Lipids and lipid-like molecules | 0.08 | | 0.023 | | | 1.46 |
| 2,3-dinor Prostaglandin E1 | Lipids and lipid-like molecules | 2.88 | | 0.025 | | | 1.08 |
| N-lactoyl-phenylalanine | Organic acids and derivatives | 0.46 | | 0.028 | | | 1.10 |
| (±)9-HpODE |  | 0.24 | | 0.029 | | | 1.37 |
| N-Acetylvaline | Organic acids and derivatives | 0.48 | | 0.029 | | | 1.34 |
| 3,3-Dimethylglutaric acid | Lipids and lipid-like molecules | 2.27 | | 0.030 | | | 1.18 |
| 2-deoxyglucose-6-phosphate | Organic acids and derivatives | 0.40 | | 0.031 | | | 1.28 |
| LPI 16:0 | Lipids and lipid-like molecules | 3.09 | | 0.033 | | | 1.40 |
| 5-Methoxyindoleacetic acid | Organoheterocyclic compounds | 0.21 | | 0.033 | | | 1.19 |
| PE (11:0/14:0) | Lipids and lipid-like molecules | 0.44 | | 0.033 | | | 1.19 |
| Genistein | Phenylpropanoids and polyketides | 0.13 | | 0.034 | | | 1.11 |
| (+/-)9-HpODE | Lipids and lipid-like molecules | 0.28 | | 0.035 | | | 1.34 |
| (±)12(13)-DiHOME |  | 0.17 | | 0.035 | | | 1.23 |
| FAHFA (22:3/20:2) | Lipids and lipid-like molecules | 2.33 | | 0.037 | | | 1.11 |
| 5-(3-chloro-4-methylanilino)-1-methyl-1H-pyrazol-3-ol |  | 0.25 | | 0.037 | | | 1.17 |
| N-Acetyl-L-phenylalanine | Organic acids and derivatives | 0.29 | | 0.039 | | | 1.30 |
| 2-Hydroxyvaleric acid | Lipids and lipid-like molecules | 2.04 | | 0.040 | | | 1.36 |
| D-Mannitol 1-phosphate | Organic oxygen compounds | 0.38 | | 0.043 | | | 1.29 |
| N-Acetyl-α-D-glucosamine 1-phosphate |  | 0.32 | | 0.044 | | | 1.12 |
| 2-(acetylamino)-3-(1H-indol-3-yl)propanoic acid |  | 0.50 | | 0.044 | | | 1.25 |
| 17(S)-HpDHA | Lipids and lipid-like molecules | 0.16 | | 0.045 | | | 1.19 |
| Lysopc 17:0 | Lipids and lipid-like molecules | 3.13 | | 0.045 | | | 1.49 |
| 2-Hydroxycaproic acid | Lipids and lipid-like molecules | 2.17 | | 0.046 | | | 1.29 |
| LPS 16:0 | Lipids and lipid-like molecules | 11.39 | | 0.048 | | | 1.84 |

Fold change= mean abundance of NR / mean abundance of R; P value was calculated by Mann-Whitney U test. VIP was calculated by PLS-DA.

1. **Supplementary Figures**

**Supplementary Figures legends:**

**Supplementary figure 1: Taxonomy structure among NR and R groups.**

1. D) α diversity analysis showed that ACE (A), Chao1 (B), Simpson (C) and Shanoon indexes (D) did not differ significantly between 2 groups.

(E-F) β diversity analysis showed that PCOA based on Bray-Curtis distance (E) and Jaccard distant (F) did not differ significantly between 2 groups.

**Supplementary figure 2: Variation of the main 12 differential bacterial species identified by Lefse among NR and NR-PD patients.** Saliva samples from NR patients at the time of PD were gathered and classified as NR-PD. The result showed that the abundance of these 12 differential species remained stable during lung cancer treatment.

**Supplementary figure 3: Correlation between each main 12 differential bacterial species.** Spearman correlation was conducted and color intensity indicated r value. * P< 0.05, ** P< 0.01.

**Supplementary figure 4: Quality control of QC samples in the negative ion mode**

(A) and negative ion mode (B). The correlation coefficients R2 of QC samples were greater than 0.9, indicating that the data quality was high.

**Supplementary figure 5: Sum of all lipids and lipid-like molecules identified in negative ion model.** (A) and positive ion model (B) was significantly associated with PD-L1 expression. P and r value were conducted via Spearman analysis.

**
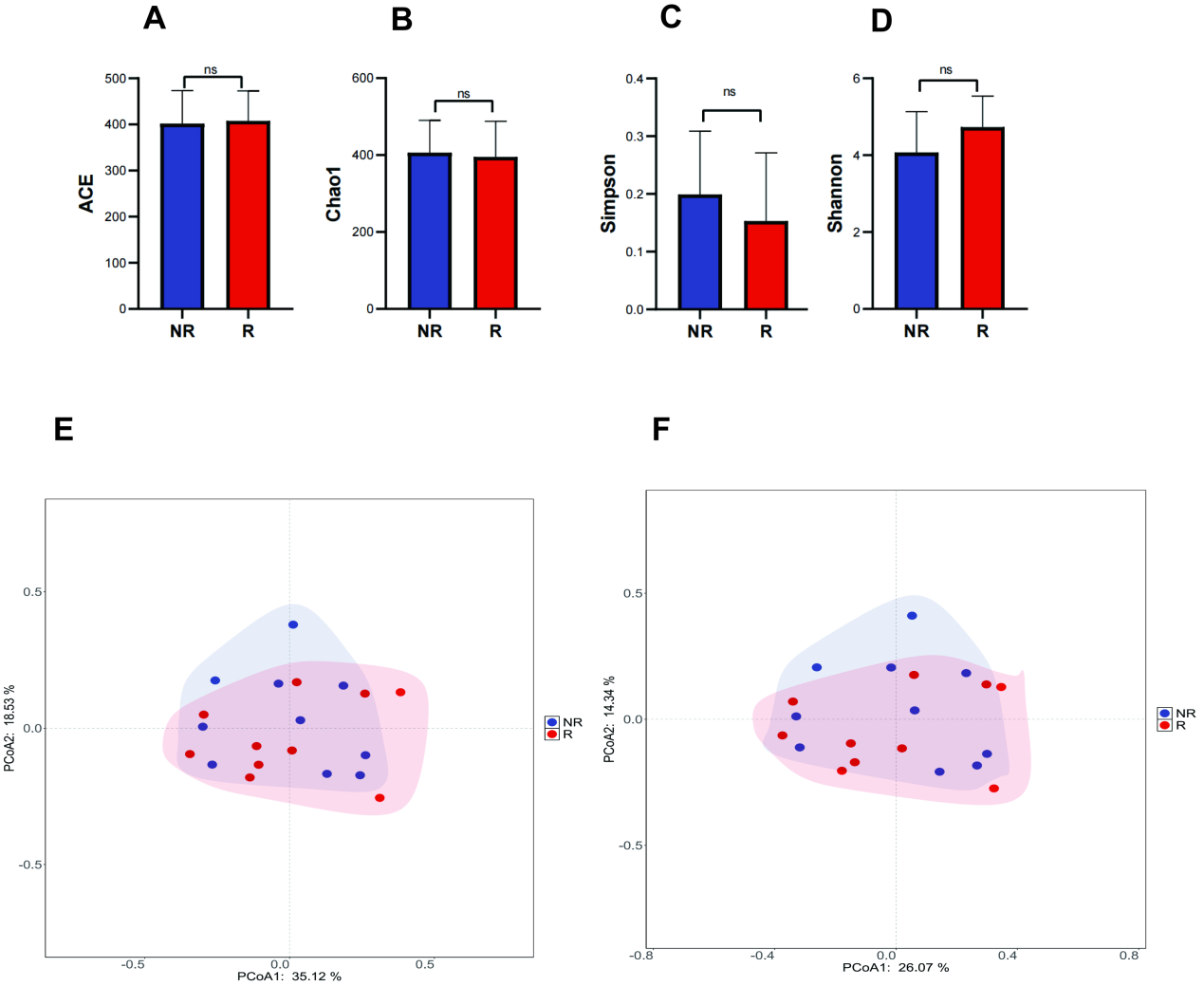
**

**Supplementary figure 1: Taxonomy structure among NR and R groups.**

1. D) α diversity analysis showed that ACE (A), Chao1 (B), Simpson (C) and Shanoon indexes (D) did not differ significantly between 2 groups.

(E-F) β diversity analysis showed that PCOA based on Bray-Curtis distance (E) and Jaccard distant (F) did not differ significantly between 2 groups.

**
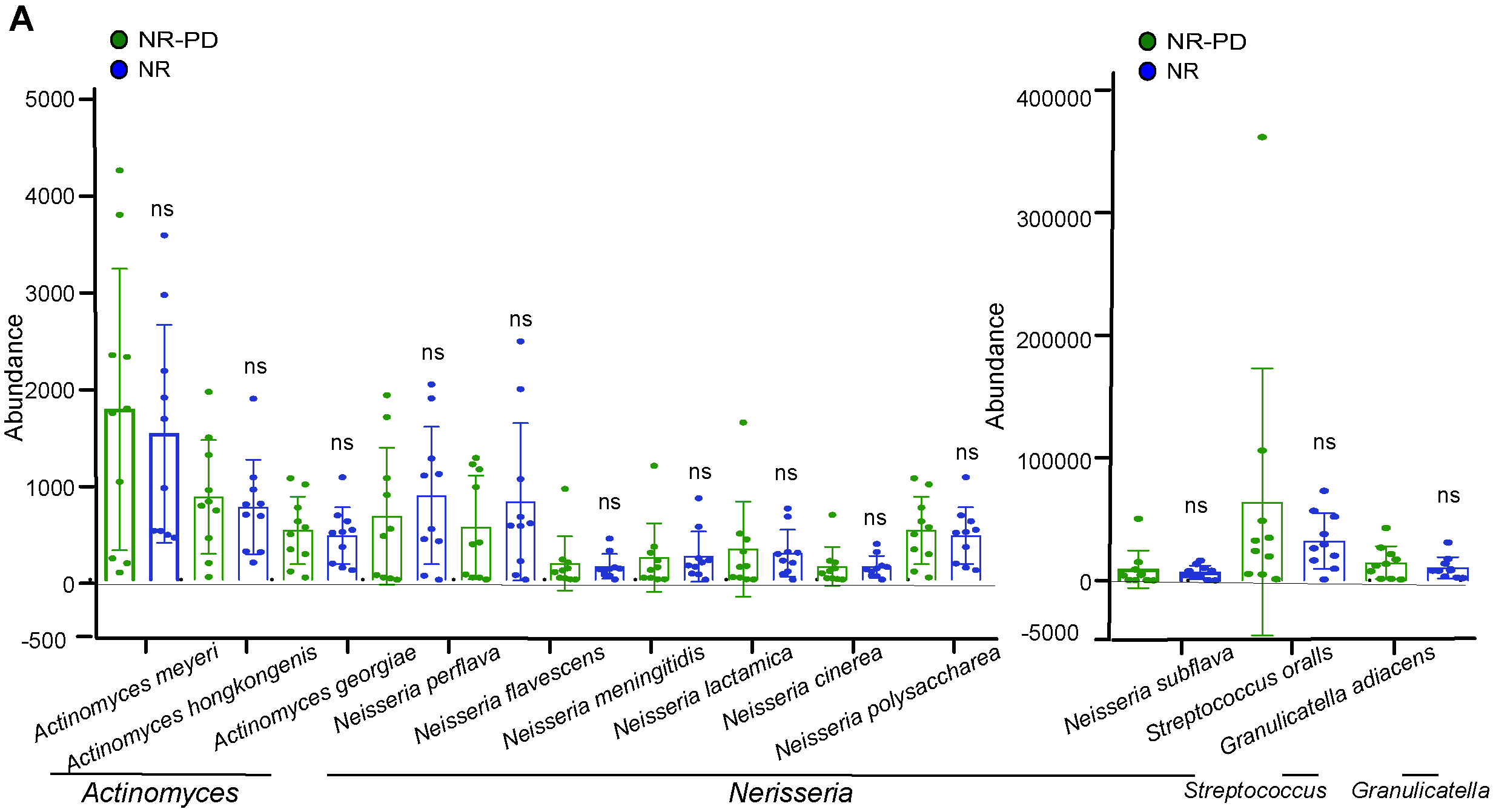
**

**Supplementary figure 2: Variation of the main 12 differential bacterial species identified by Lefse among NR and NR-PD patients.** Saliva samples from NR patients at the time of PD were gathered and classified as NR-PD. The result showed that the abundance of these 12 differential species remained stable during lung cancer treatment.

**
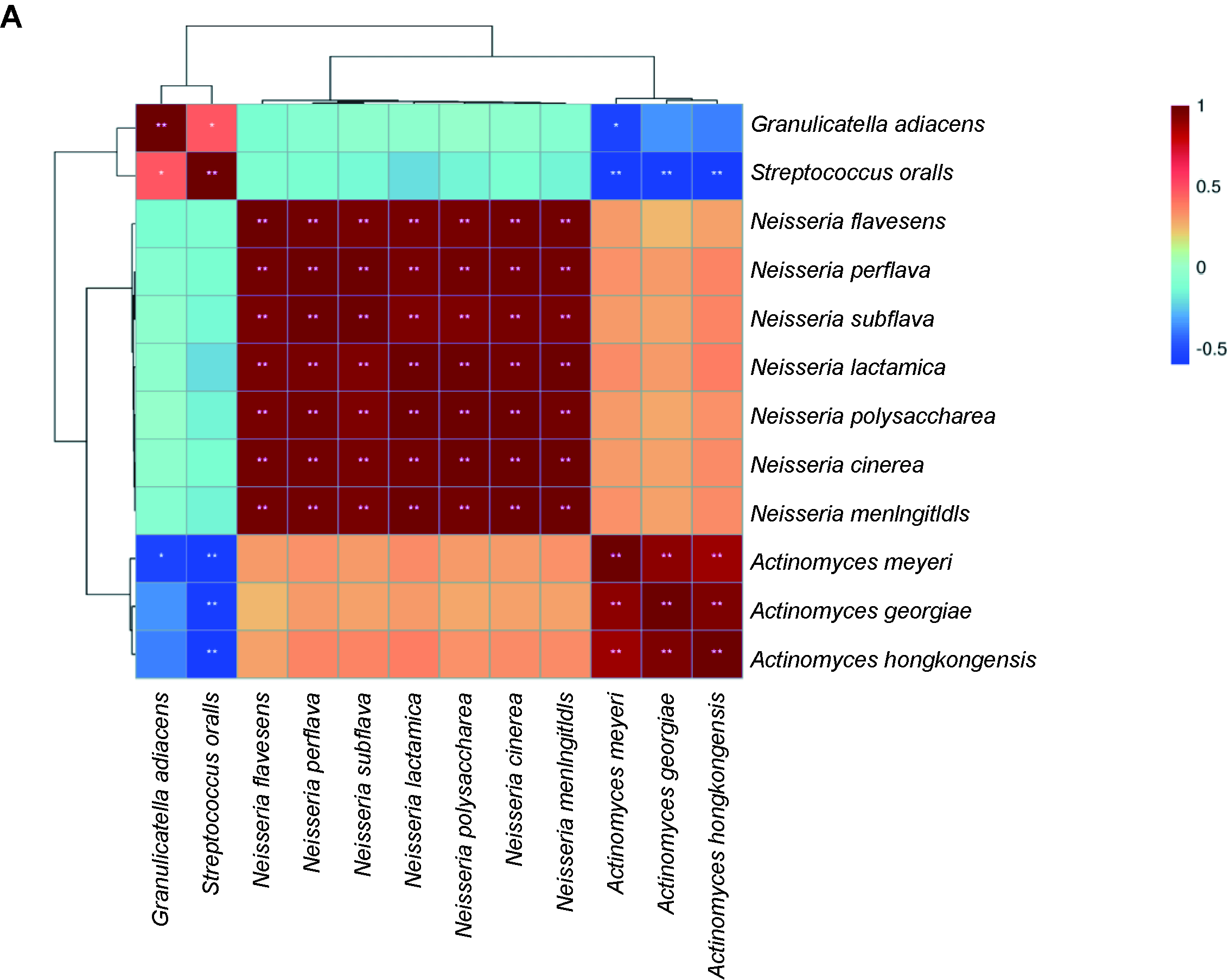
**

**Supplementary figure 3: Correlation between each main 12 differential bacterial species.** Spearman correlation was conducted and color intensity indicated r value. * P< 0.05, ** P< 0.01.

**
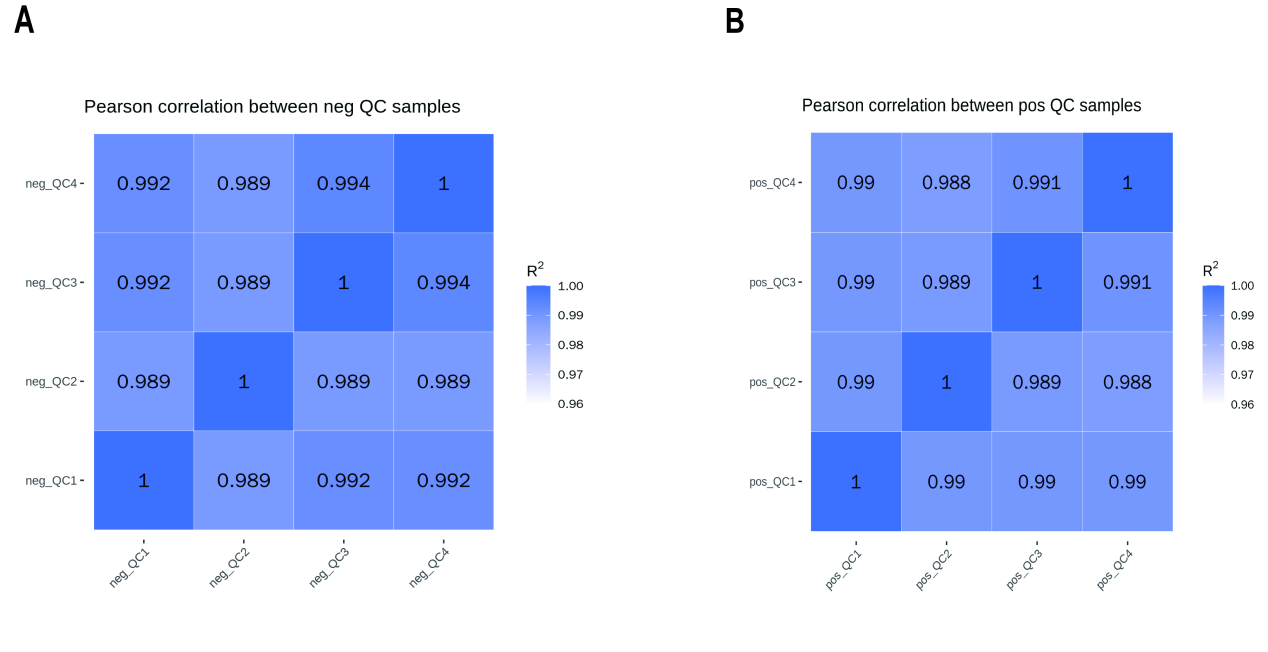
**

**Supplementary figure 4: Quality control of QC samples in the negative ion mode**

(A) and negative ion mode (B). The correlation coefficients R2 of QC samples were greater than 0.9, indicating that the data quality was high.

**
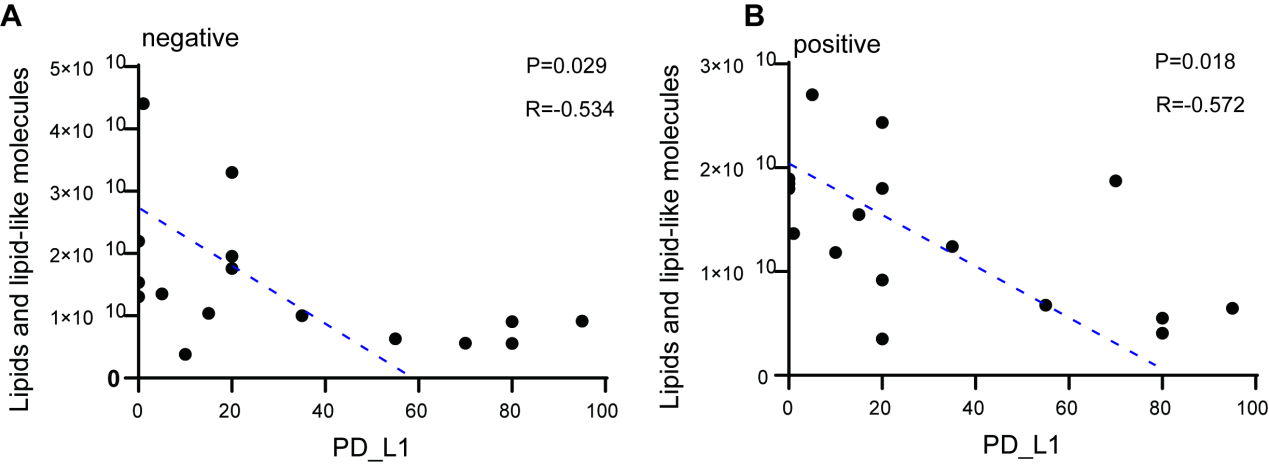
**

**Supplementary figure 5: Sum of all lipids and lipid-like molecules identified in negative ion model.** (A) and positive ion model (B) was significantly associated with PD-L1 expression. P and r value were conducted via Spearman analysis.

1. **Supplementary materials and methods**

**Statistical analysis**

The continuous variables were compared between two independent groups by Mann–Whitney U test or independent t-test. Wilcoxon signed-rank test is used for paired sample testing. The categorical variables were compared by Fisher’s exact test. p-values < 0.05 were considered statistically significant. P-value <0.05 was considered statistically significant. Spearman’s analysis was used to explore the correlation between different taxonomies and between taxonomies and metabolites.

**IHC**

The IHC was carried out as previously described. The Antigen retrieval was conducted by pressure cooker at full pressure for 3min in the EDTA Buffer pH=8.0 (ORIGENE # ZLI-9067) and then blocked in 10% FBS for 1 hour. Then the paraffin sections were incubated with the first antibodies diluted in antibodies diluent buffer (ORIGENE # ZLI-9029) at 4℃ overnight and with Horseradish Peroxidase (HRP)-conjugated secondary antibody (ORIGENE # ZLI-9017) at 37℃ for 1h. Finally, the DAB chromogenic solutions (ORIGENE # ZLI-9017) were used to detect the positive staining.

**Human Lung cancer tissue Analysis**

70 tissues were collected from patients with lung cancer receiving immunotherapy, who were subjected to surgery form Jan. 2010 to Jun. 2020 at Nangfang hospital, Southern Medical University, China. These samples were evaluated by a experienced pathologist. All of the patients had received at least one immunotherapy. These samples were detected to estimate oil-red expression. This research was approved by the Research Ethics Committee of the Nangfang hospital. The written informed consent was obtained from all the patients.

**Cell culture, Nutrients starvation**

A549 and H1650, human lung carcinoma cell lines were preserved in the Nangfang hospital, and cultured in RPMI1640 (Invitrogen #C11875500BT) supplemented with 10% FBS (Invitrogen #10099-141), 100 units/ml penicillin, and 100 μg/ml streptomycin in a humidified atmosphere containing 5% CO2. All cell lines were authenticated by STR DNA profiling (Microread Diagnostics Co., Ltd, Guangzhou, China). Nutrients starvation of cells was first cultured in total nutrient medium with 10% FBS for at least 3 days, followed by 24h starvation of 1%FBS or glucose using different nutrient deficient mediums (Chenxue Biotech #CM10043).

**T cell isolation**

All cytokines were purchased from Peprotech (Rocky Hill, NJ, USA). Human blood was obtained from healthy volunteers after informed consent and with approval by the Nanfang hospital uman Participant Ethics Committee (Ethics Approval 010558). CD3+ T cells were isolated by negative selection from freshly obtained peripheral blood mononuclear cells using human CD3+ T cell isolation kit (ImunoSep, Cat#710205). CD8+ T cells were isolated by negative selection from freshly obtained peripheral blood mononuclear cells using human CD8+ T cell isolation kit (ImunoSep, Cat#710805). Purity was confirmed with CD3E-FITC (BD Biosciences, Cat# 561806) and CD8A-FITC (Cat# CST, Cat# 85336) and analyzed by flow cytometry. Cells were cultured in RPMI 1640 medium (Sigma) supplemented with 10% heat-inactivated endotoxin-tested FCS (Biochrom GmbH, Berlin, Germany).

**T cell migration assay**

Human T cell migration was investigated by trans-well migration assays (Corning). 106 transduced T cells were placed into the upper chamber of a trans-well plate with a 3 μm pore filter. The lower chamber contained different tumour cell supernatant. To generate tumour cell supernatant, 105 A549 orH1650 lung carcinoma cells were seeded into 6-well plates and starvation for FBS or glucose for 24 h and supernatants were harvested and used for migration assay. The numbers of migrated cells in the lower chamber were quantified by FACS analysis after an incubation at 37°C for 3 – 4 hours.

**Antibodies and flow cytometry**

The surface antigens CD8, CD3E, and IFN gamma were stained with the following fluorescent labeled antibodies: anti CD8A (CST, Cat# 85336), anti-CD3E (BD Biosciences, Cat# 561806) and anti IFN gamma (CST, Cat# 8455) for 30 min at 4 °C. Cells were fixed in 1% paraformaldehyde and analyzed by flow cytometry (FACS Canto II, BD Biosciences). The antibodies:CD8a (CST, Cat# 85336), PD-L1(CST, Cat# 86163), GAPDH (CST, Cat# 2118) was used for IHC or Western blot experiments.
